# Widespread Aberrant Alternative Splicing despite Molecular Remission in Chronic Myeloid Leukaemia Patients

**DOI:** 10.3390/cancers12123738

**Published:** 2020-12-11

**Authors:** Ulf Schmitz, Jaynish S. Shah, Bijay P. Dhungel, Geoffray Monteuuis, Phuc-Loi Luu, Veronika Petrova, Cynthia Metierre, Shalima S. Nair, Charles G. Bailey, Verity A. Saunders, Ali G. Turhan, Deborah L. White, Susan Branford, Susan J. Clark, Timothy P. Hughes, Justin J.-L. Wong, John E.J. Rasko

**Affiliations:** 1 Computational BioMedicine Laboratory Centenary Institute, The University of Sydney, Camperdown, NSW 2050, Australia; u.schmitz@centenary.org.au (U.S.); v.petrova@centenary.org.au (V.P.); 2 Gene & Stem Cell Therapy Program Centenary Institute, The University of Sydney, Camperdown, NSW 2050, Australia; j.shah@centenary.org.au (J.S.S.); B.Dhungel@centenary.org.au (B.P.D.); geoffray.monteuuis@helsinki.fi (G.M.); C.Metierre@centenary.org.au (C.M.); C.Bailey@centenary.org.au (C.G.B.); 3 Faculty of Medicine & Health, The University of Sydney, Camperdown, NSW 2050, Australia; j.wong@centenary.org.au; 4 Epigenetics Research Laboratory, Genomics and Epigenetics Division, Garvan Institute of Medical Research, Darlinghurst, NSW 2010, Australia; p.luu@garvan.org.au (P.-L.L.); s.clark@garvan.org.au (S.J.C.); 5 Kinghorn Centre for Clinical Genomics Core Facility, Garvan Institute of Medical Research, Darlinghurst, NSW 2010, Australia; s.nair@garvan.org.au; 6 St Vincent’s Clinical School, Faculty of Medicine, University of New South Wales, Darlinghurst, NSW 2010, Australia; 7 Cancer Program, Precision Medicine Theme, South Australian Health & Medical Research Institute, Adelaide, SA 50000, Australia; Verity.Saunders@sahmri.com (V.A.S.); Deborah.White@sahmri.com (D.L.W.); 8 APHP, Division of Hematology, Paris Sud University Hospitals and Inserm U935 INGESTEM Pluripotent Stem Cell Infrastructure 78 Rue du Général Leclerc, 94275 Le Kremlin Bicetre, France; ali.turhan@aphp.fr; 9 School of Medicine, Faculty of Health and Medical Sciences, University of Adelaide, Adelaide, SA 5000, Australia; susan.branford@sa.gov.au (S.B.); Tim.Hughes@sahmri.com (T.P.H.); 10 Australasian Leukaemia and Lymphoma Group, Richmond, VIC 3121, Australia; 11 School of Biological Sciences, Faculty of Sciences, University of Adelaide, Adelaide, SA 5000, Australia; 12 South Australian Health and Medical Research Institute, Adelaide, SA 5000, Australia; 13 Department of Genetics and Molecular Pathology, Centre for Cancer Biology, SA Pathology, Adelaide, SA 5000, Australia; 14 School of Pharmacy and Medical Science, Division of Health Sciences, University of South Australia, Adelaide, SA 5000, Australia; 15 St Vincent’s Clinical School, University of New South Wales, Darlinghurst, NSW 2010, Australia; 16 Department of Haematology, Centre for Cancer Biology, SA Pathology, Adelaide, SA 5001, Australia; 17 Epigenetics and RNA Biology Program Centenary Institute, The University of Sydney, Camperdown, NSW 2050, Australia; 18 Cell and Molecular Therapies, Royal Prince Alfred Hospital, Camperdown, NSW 2050, Australia

**Keywords:** transcriptomic complexity, alternative splicing, intron retention, DNA methylation, epigenetics, BCR-ABL1, histone modifications, CML, cancer

## Abstract

**Simple Summary:**

This study provides new insights into the changing transcriptomic and epigenomic landscapes in chronic myeloid leukaemia (CML) patients who are receiving tyrosine kinase inhibitor (TKI) therapy (often life-long). Alternative splicing, vital for cellular homeostasis, is dysregulated in human cancers. Remarkably, we found abnormal splicing patterns despite molecular remission in peripheral blood cells of chronic-phase CML patients. This phenomenon is independent of the TKI drug used and in striking contrast to the normalisation of gene expression and DNA methylation patterns.

**Abstract:**

Vast transcriptomics and epigenomics changes are characteristic of human cancers, including leukaemia. At remission, we assume that these changes normalise so that omics-profiles resemble those of healthy individuals. However, an in-depth transcriptomic and epigenomic analysis of cancer remission has not been undertaken. A striking exemplar of targeted remission induction occurs in chronic myeloid leukaemia (CML) following tyrosine kinase inhibitor (TKI) therapy. Using RNA sequencing and whole-genome bisulfite sequencing, we profiled samples from chronic-phase CML patients at diagnosis and remission and compared these to healthy donors. Remarkably, our analyses revealed that abnormal splicing distinguishes remission samples from normal controls. This phenomenon is independent of the TKI drug used and in striking contrast to the normalisation of gene expression and DNA methylation patterns. Most remarkable are the high intron retention (IR) levels that even exceed those observed in the diagnosis samples. Increased IR affects cell cycle regulators at diagnosis and splicing regulators at remission. We show that aberrant splicing in CML is associated with reduced expression of specific splicing factors, histone modifications and reduced DNA methylation. Our results provide novel insights into the changing transcriptomic and epigenomic landscapes of CML patients during remission. The conceptually unanticipated observation of widespread aberrant alternative splicing after remission induction warrants further exploration. These results have broad implications for studying CML relapse and treating minimal residual disease.

## 1. Introduction

Characterisation of BCR-ABL1 and its constitutive tyrosine kinase activity has facilitated understanding of the pathogenesis and therapy of chronic myeloid leukaemia (CML) [1]. Tyrosine kinase inhibitor (TKI) treatment results in positive responses in 60–90% of patients with chronic-phase CML [2,3]. However, molecular mechanisms that facilitate CML maintenance and relapse remain elusive [4]. While the molecular causes of CML are well understood, measures of therapy success based on *BCR-ABL1* expression and white blood cell count are not reliable predictors of relapse.

*BCR-ABL1* overexpression perturbs the hematopoietic transcriptome [4,5]. CML progression, maintenance, and therapy may be affected by aberrant expression and alternative splicing of cancer-causing genes [6,7]. Alternative splicing is vital for cellular homeostasis and dysregulated intron retention (IR) has been associated with numerous human cancers, including leukaemia [8]. IR affects transcriptomic complexity and leads to the inclusion of premature termination codons in mature transcripts, causing nonsense-mediated decay [9,10]. However, the role of alternative splicing in CML remains unknown.

In addition to risk-associated genomic variants which predict poor outcome [11], there is evidence that epigenetic regulation affects CML pathology and therapy success [12]. Epigenetic regulation via DNA methylation and nucleosome occupancy play key roles in constitutive and alternative mRNA splicing regulation [13,14,15].

To date, no systematic transcriptomics and epigenomics analyses in CML remission samples have been conducted, neither have the effects of different therapeutic regimens on gene expression and alternative splicing been assessed. CML presents an excellent model to study epigenetically mediated transcriptomic alterations in myeloid cells that cause malignant transformation and the response to TKI treatment. New insights into this multi-layered regulatory network could provide important clues for targeted therapies used in other malignancies as well.

Here, we report novel transcriptional and epigenetic patterns in a multi-omics analysis of CML patient samples before and after effective front-line TKI treatment. Using this model, we found abnormal splicing in chronic-phase CML patients, which we confirmed in a larger independent cohort. Surprisingly, we also observed aberrant splicing patterns in complete molecular remission. This is in contrast to the normalisation of gene expression and DNA methylation patterns driven by the reconfiguration of blood cell composition. More specifically, we observed a marked increase in IR and differential exon usage in CML diagnosis and remission compared to normal samples, as well as evidence that epigenetic factors modulate splicing in CML.

## 2. Results

The goal of our work was to evaluate transcriptomic and epigenetic changes in CML patients after major or complete molecular response. We retrieved sixteen peripheral blood samples from Philadelphia-positive (Ph+) CML patients. For 10 of these patients, we also retrieved matched samples after remission. In addition, we retrieved peripheral blood from six healthy donors (Table 1). We subjected all samples to RNA sequencing and all matched diagnosis/remission samples as well as control samples to whole genome bisulfite sequencing (WGBS).

### 2.1. Heterogeneous CML Transcriptomes Converge at Remission

RNA sequencing reads mapping to the *BCR-ABL1* locus were found in diagnosis samples only (Figure S2A), confirming the results of clinical qRT-PCR-based *BCR-ABL1* quantification (Data S1). Apart from the characteristic *BCR-ABL1* fusion, we identified other recurring intra- and inter-chromosomal fusions (Figure S2A,B, Data S2) that occur in diagnosis, remission or control samples. A novel fusion transcript between the myeloid cell-specific transmembrane glycoprotein *CLEC12A* (12p13.31) and the microRNA *miR-223* host gene *MIR223HG* (Xq12) was expressed at higher levels (fold change - FC = 7.16) at diagnosis compared to remission (Figure S2C–E, Table S2).

**Figure 1:**
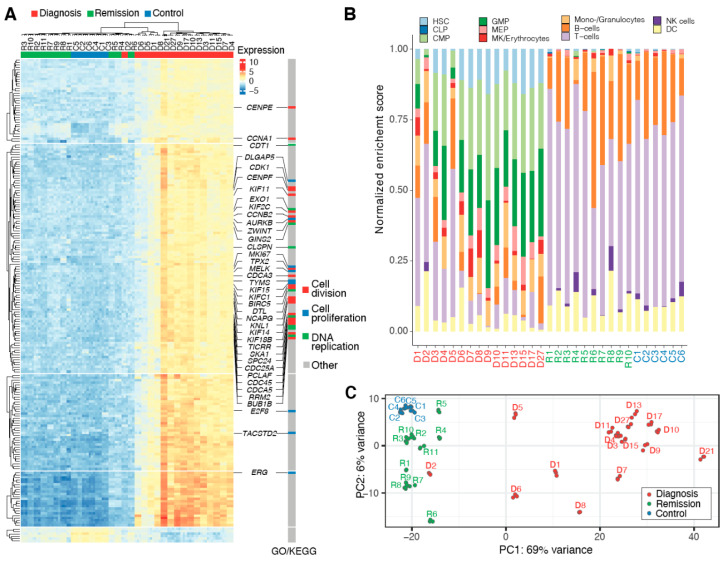
Molecular remission is reflected in transcriptomic profiles. (**A**) The Top 200 differentially expressed genes with lowest adjusted *p*-value in likelihood ratio test. (**B**) Predicted cell type enrichment in peripheral blood samples (diagnosis/red: D1–27; remission/green: R1–10; control/blue: C1–6). The stacked bar graph shows normalized cell type enrichment scores for patient and control transcriptomes based on bulk RNA sequencing data. Predictions were made with the xCell R package (github.com/dviraran/xCell) [16]. HSC—hematopoietic stem cell, CLP—common lymphoid progenitor, CMP—common myeloid progenitor, GMP—granulocyte/macrophage progenitor, MEP—megakaryocyte/erythroid progenitor, MK—megakaryocyte, NK—natural killer, DC—dendritic cell. (**C**) Principal component analysis separates diagnosis samples from the common features of remission samples and healthy controls.

For most patients, we observed a global reduction of gene expression in remission compared to diagnosis (Figure S3A). This can be attributed to the reconfigured blood cell composition, which resembles lymphoid cell-enriched normal controls (Figure 1A,B). A significant downregulation was observed in genes encoding kinases other than *BCR-ABL1* and kinase-like proteins such as *CENPE*, *CDK1*, *AURKB*, *MELK*, and *BUB1B* (Figure S4). Reduced expression at remission, similar to levels observed in control samples, also affected cyclins such as *CCNA1*, *CCNB2* and other cell cycle regulators, as well as genes related to DNA replication and repair (Figure 1A, Figure S5A). In this context, no consistent differences were noted between patients treated with imatinib or nilotinib (Figure S1).

A set of genes (*n* = 81), that are functionally associated with oxygen and bicarbonate transport (GO:0015671; GO:0015701) as well as the haemoglobin’s chaperone pathway (Biocarta), was significantly differentially expressed in all three comparisons (diagnosis vs. control, remission vs. control, and diagnosis vs. remission; Figure S5B). Overall, we observed that patients diagnosed with CML have highly heterogeneous transcriptomes. In contrast, remission transcriptomes are more alike (Figure 1C) and exhibit fewer differences in comparison to healthy control samples (Figure S3B).

### 2.2. DNA Methylation Profiles Return to Normal at Remission

**Figure 2:**
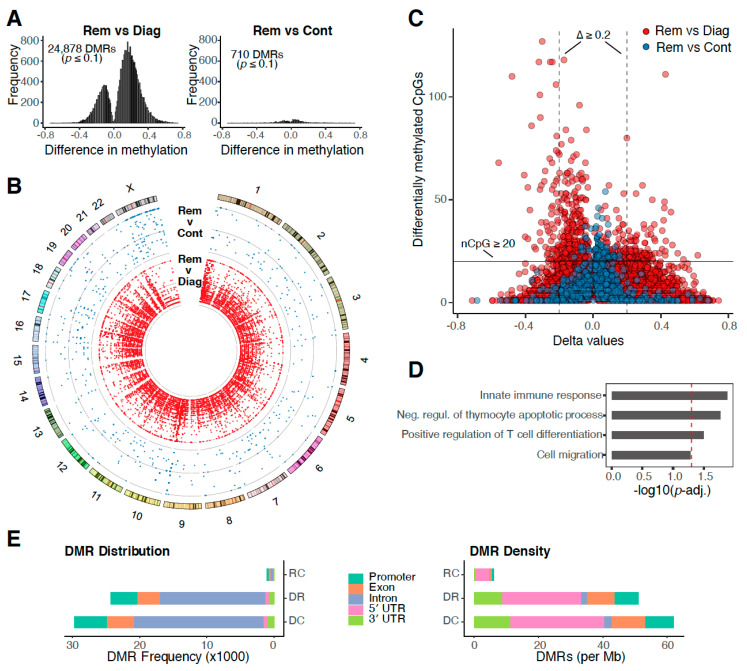
DNA methylation in chronic myeloid leukaemia (CML). (**A**) Frequency of differentially methylated regions (DMRs) based on delta values between pooled diagnosis and remission samples (left) as well as remission and control samples (right). (**B**) Circos plot illustrating genome-wide differences in the frequency of DMRs (central ring with blue scatter plot: diagnosis vs. remission, inner ring with red scatterplot: remission vs. control). This plot was generated using the Circos software [17]. (**C**) Scatterplot illustrating the number of significantly differentially methylated CpG sites per DMR and associated delta values (red points, diagnosis vs. remission; blue points, remission vs. control). (**D**) Enriched Gene Ontology (GO) terms associated with DMRs (remission vs. diagnosis) in gene promoter regions (red dashed line marks *p* = 0.05). (**E**) Distribution and density of DMRs across gene features. RC—remission vs. control, DR—diagnosis vs. remission, DC—diagnosis vs. control.

To characterise putative epigenetic causes for differential gene expression in CML patients prior to and after successful TKI treatment, all matched diagnosis/remission samples as well as all control samples were subjected to WGBS. The analysis revealed almost 25,000 differentially methylated regions (DMRs) in the genomes of diagnosis and remission samples (Figure 2A, left). Vast DNA methylome differences were found in every matched patient sample (Figure S6) and across all chromosomes (Figure 2B). In contrast, only 710 DMRs were observed between remission and control samples (Figure 2A, right). Indeed, multidimensional scaling showed that DNA methylation profiles of remission samples are closer to those from healthy donors rather than to matched diagnosis samples (Figure S7).

DMRs between diagnosis and remission samples contain, on average, a larger number of differentially methylated CpG sites (Figure 2C). This is consistent with previous reports that suggest an increase in CpG site methylation during CML progression that affects tumour-suppressor genes and regulators of cell proliferation [12]. DMRs are associated with processes of the innate immune response, apoptosis, cell differentiation and cell migration (Figure 2D).

Our results show that DNA methylation returns to normal levels after successful TKI treatment. Most DMRs can be found in intronic regions, however, the highest density of DMRs was observed in 5′ untranslated regions (Figure 2E), suggesting a key role for differential DNA methylation in orchestrating CML gene expression regulation.

### 2.3. Aberrant Alternative Splicing Distinguishes Remission Samples from Healthy Controls

To assess whether major or complete molecular remission also affects gene isoform expression, we conducted a systematic analysis of the five major forms of alternative splicing (Figure S8A). In a pairwise comparison of alternative splicing event frequencies between matched diagnosis/remission samples, no consistent trend towards an increase or decrease in either of the two conditions was found (Figure S8B). This suggests that in contrast to gene expression profiles, splicing patterns remain atypical after major or complete molecular remission.

**Figure 3:**
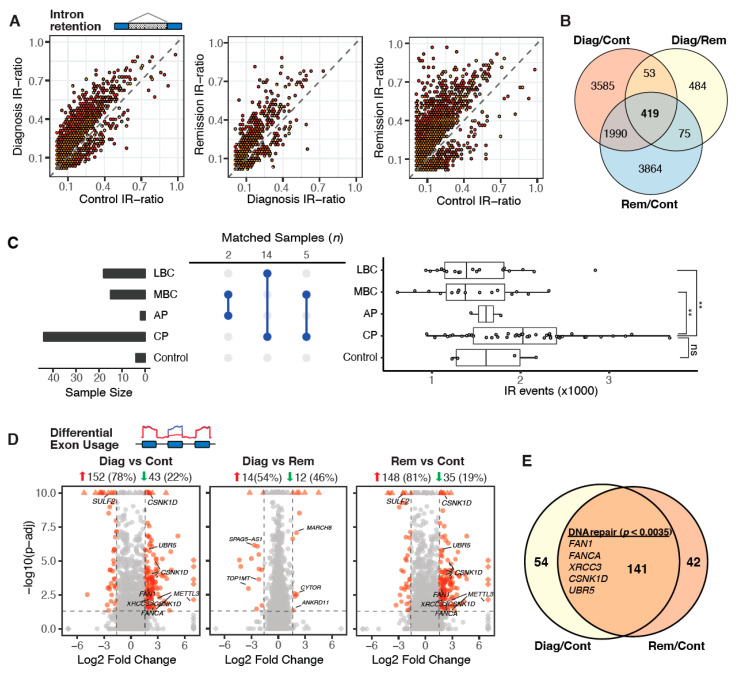
Global alternative splicing patterns are similar despite major molecular remission. (**A**) Scatterplots illustrating differential IR events between pooled diagnosis/remission/control samples binned into hexagons to avoid overplotting (yellow: *adj.-p* ≤ 0.05; red: *adj.-p* ≤ 0.01). (**B**) Venn diagram showing intersections of mutually differentially retained introns. (**C**) Dynamics of IR across CML phases. CML patient data from Branford et al [11]. was analysed for the occurrence of IR events. Left: Sample sizes for Control, CP: Chronic Phase, AP: Accelerated Phase, MBC: Myeloid Blast Crisis and LBC: Lymphoid Blast Crisis samples. Middle: Sample numbers matched from different phases. Right: Boxplot of IR frequencies in samples from different CML phases. Mann–Whitney U test; ** (*p* < 0.01), ns (*p* > 0.05). (**D**) Differential exon usage in pooled diagnosis vs. control (left), diagnosis vs. remission (middle) and remission vs. control samples. Numbers following the red arrows indicate how many upregulated DUEs in the first of the two conditions compared. Numbers following the green arrows indicate how many downregulated DUEs in the first of the two conditions compared. (**E**) Intersection of differentially used exons in diagnosis and remission samples compared to controls (the top-enriched GO term and associated genes are listed in the middle).

Alternative splicing analysis of the Cancer Genome Atlas (TCGA) cohort by Dvinge et al [8]. revealed that the frequency of IR events is consistently higher in acute myeloid leukaemia compared to normal controls [8]. Although mean IR frequencies are also higher in CML samples (diagnosis, *µ* = 3382; remission, *µ* = 3484) compared to healthy donors (*µ* = 2822), high inter-donor variability led to differences in IR frequencies that did not reach significance (Figure S8C). However, when we subsampled the RNA sequencing data to facilitate a uniform sequencing depth for comparison [18], we observed a clear separation with significantly increased IR frequencies at diagnosis and remission compared to controls (Figure S9). Moreover, we found that the IR ratios are consistently higher in diagnosis and remission samples compared to healthy controls (Figure 3A). Differences between diagnosis and remission samples were less pronounced (Figure 3A and Figure S10). While IR profiles are unsuitable for patient stratification (Figure S11), differentially retained introns between diagnosis and remission samples are, similar to differentially expressed genes, associated with cell cycle regulators as well as genes related to DNA replication and repair (Figure S12). However, in remission, IR primarily affects genes involved in splicing processes (Figure S12), suggesting that aberrant splicing persists in remission due to auto-regulatory processes. Overall, IR profiles are highly dynamic in CML, which becomes apparent when considering the large overlap of differentially retained introns (*n* = 419) in all three comparisons (Figure 3B). We confirmed these results with an independent dataset of 59 CML patients from Branford et al. [11], which includes samples from various stages of disease progression, and from 4 healthy controls (Figure 3C). The number of IR events in this cohort was highly variable. While there are on average more IR events in chronic phase compared to healthy controls, the number drops significantly at blast crisis (Figure 3C, right).

Examining differential exon usage in our samples, we observed a similar trend, although not as marked as observed in differential IR, i.e., both diagnosis and remission samples exhibit a larger number of differentially used exons (DUEs) compared to normal controls. Most of these DUEs are upregulated (diagnosis, 78%; remission, 81%; Figure 3D). A large fraction of the DUEs at diagnosis (72%) remain differentially expressed at remission as well. Some genes of these mutual DUEs are associated with DNA repair mechanisms (top-enriched GO term; Figure 3E). Only 26 exons in total are differentially used in diagnosis vs. remission samples (Figure 3D).

### 2.4. Modulation of IR Levels Supports Lineage-Specific Gene Expression in CML

**Figure 4:**
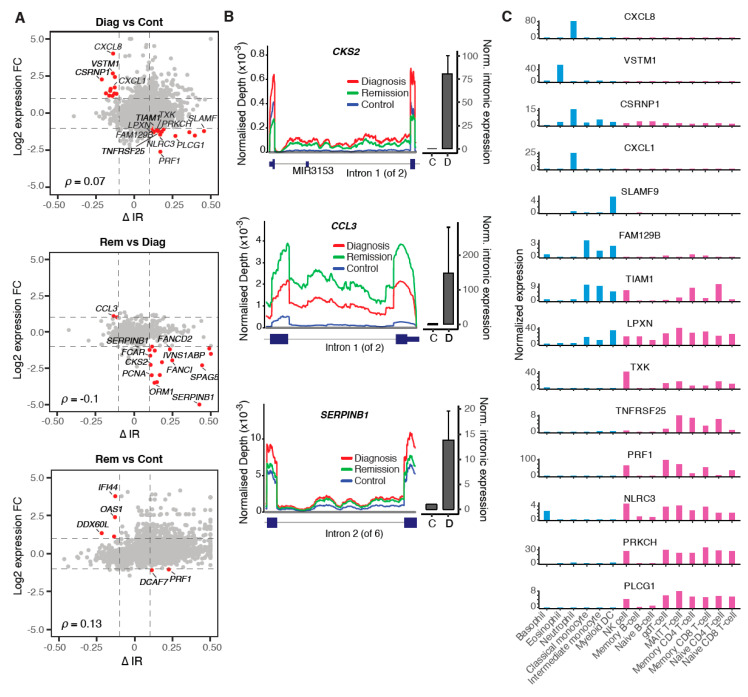
Differentially retained introns inhibit the expression of cell cycle regulators following major molecular remission. (**A**) Scatterplots illustrating the relationship between differentially retained introns (∆IR) and differential gene expression (log2 expression fold change). Introns (|∆IR| ≥ 0.1) with inverse relationship to host gene expression (|log(FC)| ≥ 1) are highlighted in red. (**B**) Coverage plots of selected differential IR events (left) and qRT-PCR validation of differential IR between diagnosis and control (right). qRT-PCR validation was performed in triplicates on independent diagnosis samples and results were normalised to *B2M* expression. D—diagnosis, C—control. (**C**) Blood cell type expression of genes with inverse IR/gene expression relationships (diagnosis vs. control; blue—myeloid, magenta—lymphoid cells). The data were retrieved from the Human Protein Atlas (v19.3; http://www.proteinatlas.org) [24].

IR is accepted as a mechanism of post-transcriptional gene regulation triggering nonsense-mediated decay due to the presence of premature termination codons within the retained introns [19,20]. We analysed the relationship between IR and gene expression changes, but overall, the two variables did not significantly correlate (Figure 4A). However, many of the genes, whose expression does anti-correlate with the retention of their introns, are involved in cell cycle regulation (e.g., *CCL3*, *CKS2* and *SPAG5*) and DNA replication/repair (e.g., *FANCI*/*FANCD2* and *PCNA*) (Figure 4A).

We selected three differentially retained introns of cell cycle regulator genes for experimental validation (*CKS2* intron 1, *CCL3* intron 1 and *SERPINB1* intron 2). Based on the RNA sequencing data analysis, all three introns have an increased expression and higher IR ratio at diagnosis compared to healthy controls (Figure 4A). We confirmed these observations via qRT-PCR (Figure 4B). The IR ratios of *CKS2* intron 1 and *SERPINB1* intron 2 increase further at remission, resulting in a reduction of host gene expression, while the IR ratio of *CCL3* intron 1 is reduced at remission and associated with an increase in gene expression (Figure 4A,B).

Interestingly, some instances of inverse relationships between IR and gene expression affect lineage-specific genes (Figure 4C). For example, the chemokine Interleukin-8 (*CXCL8*), which is predominantly expressed in neutrophils, is upregulated at diagnosis. This could be attributed to the increased myeloid progenitor cell abundance (Figure 1B), but also to the reduced IR levels in *CXCL8* mRNA (Figure 4A). In control samples, the IR levels increase from 12.9% to 26.7% in *CXCL8* (intron 2), while gene expression is reduced 4-fold. *CXCL8* promotes CML cell proliferation [21] and previous reports suggest that *CXCL8* expression is modulated both by *BCR-ABL1* expression (causing an increase) and TKI treatment (causing inhibition of *CXCL8*) [22].

In contrast, *PRKCH* is downregulated at CML diagnosis along with increased IR levels (Figure 4A). *PRKCH* is a serine-threonine kinase that regulates hematopoietic stem cell function and is specifically expressed in lymphoid cells. Notably, high *PRKCH* expression has been associated with poor prognosis in acute myeloid leukaemia [23].

These observations suggest that lineage-specific expression of drivers of cell proliferation and hematopoietic cell differentiation can be mediated through IR level changes in CML.

### 2.5. Multiple Regulatory Mechanisms Modulate Intron Retention in CML

**Figure 5:**
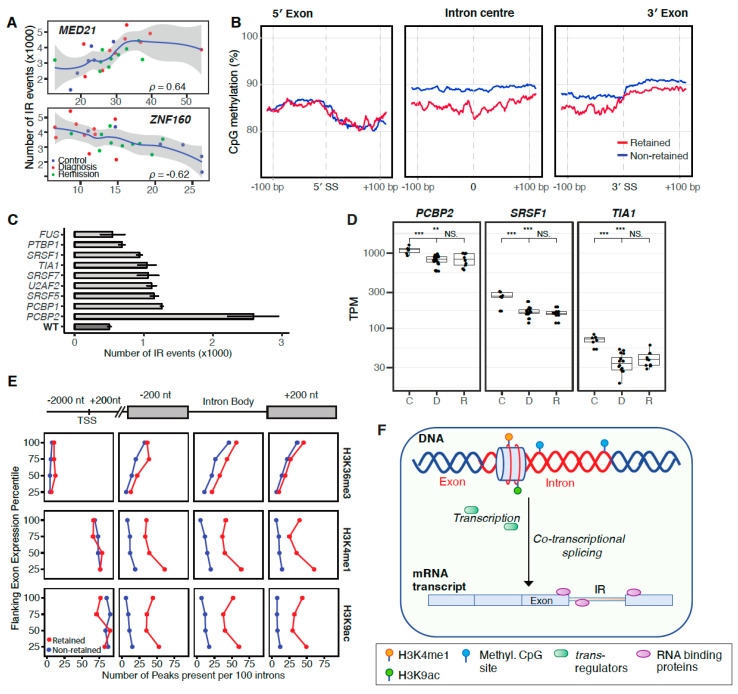
Regulation of IR in CML. (**A**) Two genes that most strongly correlate/anti-correlate with IR frequencies (Spearman correlation). (**B**) CpG methylation around intron splice sites (± 200 bp) and in the centre (200 bp) of retained- (red) and non-retained introns (blue) in CML diagnosis samples. General linear hypothesis test; *p* < 2.2 × 10^−16^ (*p-adj.* < × 1 × 10^−10^). (**C**) The effect of RNA binding proteins on IR frequency. RNA-seq data of small hairpin RNA (shRNA)-mediated knockdown of indicated RNA binding proteins in K562 cells was retrieved from the ENCODE project (encodeproject.org). WT—wild type. (**D**) Expression of RNA binding proteins with enriched binding motifs near frequently retained introns [27]. TPM - transcripts per million mapped reads; C—control; D—diagnosis; R—remission; NS.—non-significant, ** *p* < 0.01, *** *p* < 0.001. (**E**) Histone modifications with increased numbers of peaks near splice sites of retained introns (red: retained; blue: non-retained introns). The analysis was repeated for each expression quartile to exclude the possibility that increased histone modifications are associated with gene expression. (**F**) Possible mechanisms of IR regulation in CML discussed in the text.

Characteristics of the retained introns in our CML cohort are in accordance with previously described attributes, such as their relatively short length (Figure S13) and higher guanine-cytosine (GC) content (Figure S14). While these are intrinsic features of IR, we sought potential *trans*-regulators and found that the gene that most strongly correlates (*p* = 0.64) with IR frequencies is *MED21* (Mediator Complex Subunit 21; Figure 5A). *MED21* regulates gene transcription by interacting with RNA polymerase II [25]. RNA polymerase II elongation rates influence splice site recognition and IR [15]. Interestingly, the expression of *ZNF160*, encoding a Zinc Finger Protein and repressor of transcription, strongly anti-correlates (*p* = −0.62) with IR frequencies (Figure 5A).

We also examined expression changes in components of the super-elongation complex (SEC), the negative transcription elongation factor (*NELF*), the 5,6-dichloro-1-β-d-ribofuranosylbenzimidazole (DRB) sensitivity-inducing factor (*DSIF*), and others. A persistent upregulation, at diagnosis and remission, was observed for the eleven-nineteen Lys-rich leukaemia (ELL) family member *ELL2* (Figure S15), which is a factor involved in suppressing transient pausing of Pol II [26]. The negative elongation factors *NELFB* and *NELFCD* are consistently downregulated at diagnosis and remission (Figure S15). A coordinated interplay between p-TEFb, NELF and DSIF is required for the release of Pol II from pausing sites to start productive elongation, which might be impacted by reduced NELF levels, leading to aberrant splicing [26].

We have previously shown that DNA methylation regulates IR in myeloid cells [15]. To confirm this mode of regulation in our CML cohort, we compared CpG methylation around the splice sites (±200 bp) and in the centre (200 bp) of retained and non-retained introns. We confirmed decreased DNA methylation levels near the 3′ splice junction and within the body of retained introns (Figure 5B). Similar patterns were observed in remission and control samples, suggesting that this mode of IR regulation is also present in lymphoid cells (Figure S16).

To further investigate the role of epigenetics in the regulation of IR in CML, we retrieved RNA-seq and chromatin immunoprecipitation sequencing (ChIP)-seq data of the K562 CML cell line from ENCODE (encodeproject.org). We analysed transcriptomics data of RNA binding protein knockdown experiments and observed significantly increased IR occurrences with the most drastic IR increase (~5 fold) following Poly(RC) Binding Protein 2 (*PCBP2*) knockdown (Figure 5C). All genes with enriched binding motifs near frequently retained introns [27] were downregulated at CML diagnosis, except for *SRSF5* (Figure S17). Three of these RNA-binding proteins, including *PCBP2*, remained downregulated in remission (Figure 5D).

To identify additional potential explanations for the dysregulation of IR in CML, we analysed histone modifications in K562 cells. While higher levels of H3K36 trimethylation have previously been associated with IR [28,29], we found only marginal differences in H3K36me3 levels in retained versus non-retained introns. Increasing numbers of H3K36me3 peaks around introns were observed in more highly expressed genes (Figure S18). However, we found that monomethylation and acetylation of histone H3 lysines 4 and 9 respectively (H3K4me1 and H3K9ac), were significantly enriched near splice sites of retained introns and within their intron bodies in contrast to non-retained introns (Figure 5E). While a significant enrichment of H3K9ac has been found to co-occur at many gene regulatory elements in mouse embryonic stem cells [30], here, we established a strong association of H3K9ac with IR. Elevated levels of H3K4 methylation in association with IR have been previously reported by Zhou et al. [28].

In summary, our analysis of CML patient transcriptomes revealed aberrant alternative splicing both at diagnosis and remission. Elevated IR ratios in CML can be explained by multiple regulatory mechanisms including the regulation of RNA Pol II elongation, demethylation of CpG sites, histone modifications and RNA binding proteins (Figure 5F). The TKI treatment itself seems to have no effect on IR (Figure S19).

## 3. Discussion

In this study, we analysed epigenomics and transcriptomics data from patients diagnosed with chronic-phase Ph+ CML and matched remission samples after effective treatment with TKIs. Thereby, we generated novel insights into the molecular responses to TKI therapy beyond the known reduction in *BCR-ABL1* expression.

We have identified recurring fusion transcripts in patient samples, including an uncharacterised fusion between the cell surface marker C-type lectin domain family 12 member A (*CLEC12A*; 12p13.31) and the *MIR223* host gene (Xq12). The expression of *CLEC12A*::*MIR223HG* is low in remission and control samples but significantly increased at diagnosis. Therefore, it warrants further investigation to determine its relevance in CML. Other non-specific intra-chromosomal fusions include *EEF1DP3-FRY*, *EIF4E3-FOXP1*, *KANSL1-ARL17B* and the mitochondrial fusion *ND6-TE* (Supplementary Figure S2A). An increasing number of mitochondrial mutations and fusions have been described as a consequence of increased reactive oxygen species during ageing [31], however, the age range (21–70 years) of *ND6-TE*-positive patients in our cohort does not support an age-related occurrence of this fusion.

Gene expression profiles of our CML remission samples are more similar to those of healthy donors, likely due to the change in cell composition and the loss of *BCR-ABL1* clones. Consistent with previous studies, we found cell cycle regulators among the most aberrantly expressed genes in the diagnosis samples. For example, cyclins A1 and B2 (*CCNA1*, *CCNB2*) were among the most differentially expressed genes, with overexpression at diagnosis and low expression at remission (comparable to expression levels observed in healthy controls). While cyclin D2 (*CCND2*) has previously been characterised as a *BCR-ABL*-dependent mediator of cell proliferation in hematopoietic cells [32], marginal changes in *CCND2* (FC = 0.87, *p* = 0.04) expression suggest that other cyclins such as *CCNA1* (FC = 12.3, *p* = 5.4 × 10^−53^) and *CCNB2* (FC = 7.2, *p* = 9.2 × 10^−55^) were among the more potent regulators of cell proliferation in this cohort of CML patients.

The most surprising result of our study was that, in contrast to gene expression, alternative splicing patterns did not return to normal in CML remission samples. At diagnosis, the peripheral blood contains a large proportion of myeloid progenitor cells and stem cells, which are known to express high numbers of intron-retaining transcripts compared to other blood cell types [19,20,27,33]. In remission, however, where myeloid progenitor cells are diminished, aberrant splicing seems to affect the lymphoid-enriched cell populations. It is important to note that, similar to myeloid lineages, lymphoid cells undergo dynamic IR programs during differentiation and upon activation [34,35]. For example, IR transcript expression is highest in naïve or resting B-cells and decreases when B-cells undergo affinity maturation [35]. Likewise, IR is prevalent in resting CD4+ T-cells and dramatically reduced upon activation [34]. Interestingly, it has been shown that imatinib inhibits T-cell proliferation and activation [36,37] and that TKIs impair B-cell immune responses in CML [38]. In this context, immunosuppressive effects have been observed in CML patients in remission, possibly due to TKI off-target effects [39,40,41].

Shen et al [42]. have shown that alternative splicing events can be used to construct predictors for patient survival, that outperform gene expression-based predictors in multiple cancers [42]. Splicing-based prognostic markers have already been found for several cancer types [42,43]. Therefore, a splicing-based predictor for TKI cessation success is not just desirable but an achievable prospect, once clinical data of trial TKI cessation together with remission transcriptomics data become available.

Our results were consistent across patients and different specific TKI treatments. It is known that the cell composition of the peripheral blood changes in CML—with myeloid progenitor cell-enrichment at diagnosis and mature lymphoid cell-enrichment at remission. The latter is very similar to the cell composition observed in the peripheral blood of healthy individuals (Figure S5). Therefore, epigenomic and transcriptomic differences between diagnosis and remission may be expected. However, the persistent alternative splicing perturbations in remission that do not resemble patterns observed in healthy controls are remarkable.

We found new evidence for epigenetic regulators of alternative splicing, such as reduced DNA methylation and increased H3K4me1 and H3K9ac marks around retained introns. We have previously shown that DNA methylation can regulate IR in myeloid cells and during terminal granulopoiesis [15,44]. In CML, reduced DNA methylation in the intron body and 3ʹ splice site is associated with IR (Figure 5B). A role for the promoter and enhancer mark H3K4 mono-methylation in gene repression has been identified previously [45], however, H3K4me1-dependent alternative splicing has not been described before. H3K4me1 and H3K9ac are present at the promoters of both intron-retaining and non-retaining genes but they can be predominantly found near splice sites and within bodies of retained introns in contrast to non-retained introns (Figure 5E). While both histone marks seem indicative of active transcription (Figure 5E), by regulating Pol II recruitment and elongation rates [45,46] around splice sites, they could promote IR in CML. IR regulation via H3.3K36me3-specific readers, such as previously shown in HeLa cells [29], seems less relevant in CML patients.

In summary, it seems unlikely that the observed patterns are caused by persisting leukemic clones. Most patients in this cohort were in complete molecular remission, and some in major molecular remission. Aberrant splicing, however, was widespread at remission in all patients. While the effect of persisting leukemic clones on aberrant splicing cannot be entirely ruled out, our evidence suggests that this is unlikely to be the case. Multiple recent studies have shown that aberrant IR in cancer can be triggered by epigenetic changes, splicing factor dysregulation and changes in transcription elongation [47]. We confirmed these observations in our analysis of CML patient samples and K562 cells. While we could not find evidence for direct TKI-induced aberrant splicing, the observed patterns of high IR in remission samples could also be an indication of TKI-induced immunosuppression reflected by an enrichment in naïve and resting B- and T-cell populations.

While our study provides novel insights into the changing transcriptomic and epigenomic landscapes of CML patients during remission, the implications of our results for CML relapse have yet to be elucidated.

## 4. Materials and Methods

### 4.1. Patient Samples

We retrieved nine diagnostic specimens (total leukocytes from peripheral blood collected in ethylenediaminetetraacetic acid (EDTA) from patients enrolled in the RESIST (1 imatinib; ACTRN12610000055000), ENESTxtnd (4 nilotinib, NCT01254188) and PINNACLE (4 nilotinib + pegylated interferon, ACTRN12612000851864) trials, with matched remission specimens (major or complete molecular response at 12 or 24 months). In addition, nine CML patient samples (7 diagnostic and 2 matched remission) from the French Persistem study were obtained. All patients were diagnostically tested for the presence of the *BCR/ABL1* gene fusion. Most patients also had a bone marrow cytogenetic analysis at diagnosis, confirming only the reciprocal chromosomal translocation t(9;22) (q34;q11) characteristic for CML.

For the validation of IR events and fusion transcripts, we retrieved further matched diagnosis/remission samples from the TIDELII trial (20 imatinib, ACTRN12607000325404). RNA was isolated from mononuclear cells (from peripheral blood collected in lithium heparin) which had been cryopreserved and subsequently thawed into TRIzol (Invitrogen, Carlsbad, CA, USA). All studies were approved by the institutional review boards (HREC protocol No: 131015 (on 31 Oct 2013), 081211(on 15 Dec 2008), and 101010 (on 18 Nov 2010), —Royal Adelaide Hospital; Int. 100912—Inserm internal ethical committee, 2012) and are in accordance with the Declaration of Helsinki. Patients provided written informed consent.

### 4.2. RNA Isolation and mRNA-seq

Total RNA was isolated from diagnosis, remission and control samples (PBMCs) using Trizol according to the manufacturer’s protocol. For mRNA sequencing, poly-A-enriched mRNA libraries were prepared from 1 μg of total RNA using the TruSeq Stranded RNA sample prep kit (Illumina, San Diego, CA, USA), prior to paired-end sequencing using the HiSeq 2500 platform. RNA sequencing was performed in triplicates for each sample.

### 4.3. mRNA Sequencing Data Analysis

Paired-end RNA-sequencing reads (125 nt) were trimmed and mapped to the human reference genome hg38 using STAR v2.7 [48]. Quality control of raw and mapped sequencing reads was performed using FASTQC v0.11.5 (github.com/s-andrews/FastQC), RSeQC v2.6.4 [49] and multiqc v1.2 [50]. Putative confounders and batch effects were excluded using principal components analyses (Figure S1). STAR-FUSION v1.4.0 [51] was used for the identification of fusion genes and Fusion Inspector (FusionInspector.github.io) for *in silico* validation of the predicted gene fusions. Gene expression levels specified as transcripts per million (TPM) were determined using Salmon 0.14.1 [52].

General statistics on alternative splicing events were determined using rMATS v3.2.4 [53]. We used our IRFinder algorithm (v1.2.0) for the detection of IR events in introns extracted from an Ensembl gtf file (regions between two adjacent exons) [27]. IRFinder estimates the abundance of IR by computing the ratio between gene transcripts retaining an intron and the sum of all transcripts of the respective gene (more information is provided in File S1).

Differentially used exons were determined using the R Bioconductor package DEXSeq [54]. Differential exon usage (DEU) = changes in the relative usage of exons:

transcripts_with_exon_/(transcripts_with_exon_ + transcripts_without_exon_) (1)

### 4.4. IR Validation

Extracted RNA (2 µg) was treated with DNAse Turbo (Invitrogen, Carlsbad, CA, USA) and reverse transcribed into cDNA using oligo(dT) priming and Superscript III (ThermoFisher, Waltham, MA, USA), according to the protocol supplied by the manufacturer. For each sample, a corresponding control without reverse transcriptase was used to assess DNA contamination. For qPCR, cDNA templates were amplified, and the C_t_ values were quantified with SYBR Green Master Mix (ThermoFisher). Beta-2 microglobulin (*B2M*) was used as the normalisation control for cDNA input. Experiments were performed using the CFX96 Real-Time PCR System (BIORAD, Hercules, CA, USA). The list of primers is provided in Table S1.

### 4.5. Whole Genome Bisulfite Sequencing (WGBS)

WGBS libraries were prepared following Illumina’s “Whole-Genome Bisulfite Sequencing for Methylation Analysis” protocol. Briefly, 1 μg of genomic DNA was spiked with 0.5% unmethylated lambda DNA and sonicated to generate fragments of size between 150 to 300 bp. Library preparation was performed using Illumina’s Paired-end DNA Sample Prep Kit (discontinued, Illumina, San Diego, CA, USA) according to the manufacturer’s protocol. The size-selected libraries were then subjected to bisulfite conversion as previously described [55]. Adaptor-ligated bisulfite-treated DNA was enriched by 10 cycles of PCR amplification using the PfuTurbo Cx Hotstart DNA Polymerase (Stratagene, La Jolla, CA, USA). Qualitative and quantitative checks of the libraries were performed using Agilent’s High sensitivity DNA kit (Agilent, Santa Clara, CA, USA) and KAPA Library quantification kit (KAPA Biosystems, Wilmington, MA, USA). Three lanes of paired-end 100 bp sequencing was performed for each of the libraries on the Illumina HiSeq2500 platform using the TruSeq v3 cluster kits and SBS kits (Illumina) to achieve coverage ranging between 25 × and 30 ×.

### 4.6. WGBS Data Analysis

Reads were processed and aligned to the human (hg38) reference genome using Meth10X (github.com/luuloi/Meth10X) [56]. In short, the Meth10X pipeline takes raw reads in fastq format and trims the adaptors, which are then aligned to the human reference genome using bwa-meth (github.com/brentp/bwa-meth). The generated bam files were marked with duplication and merged if necessary. Estimation of the duplication rate, coverage bias (genomic features) and methylation bias in reads was carried out to provide quality control. All the metrics, such as percentage of unmapped/mapped read metrics, mapping quality distribution, GC content distribution, insert size distribution and coverage distribution, were generated by Qualimap 2 [57] for further evaluation of the alignment. Finally, a count table and bigwig files of methylated and coverage at each CpG site in the genome was constructed for all samples. Differentially methylated regions (DMRs) were identified using MethPipe [58] with a count table of all samples as input. The analysis of differential methylation and IR is described in the File S1.

### 4.7. ChIP-seq Data Analysis

ChIP-seq data of histone modifications in K562 cells was retrieved from ENCODE (encodeproject.org) and further processed as described in the File S1.

### 4.8. Statistical Analyses

All statistical analyses were performed in R v.3.6.2 (www.r-project.org). The Wald test implemented in the R package DESeq2 [59] was used for the identification of statistically significant differential alternative splicing events and differential gene expression analysis. *p*-values were adjusted for the false discovery rate using the Benjamini-Hochberg procedure. Functional enrichment analysis of differentially expressed genes and genes with differentially retained introns was performed using DAVID 6.7 [60] with the human genome and the set of expressed genes respectively, used as a background.

### 4.9. Data Availability

The raw sequencing data (fastq) have been deposited at Gene Expression Omnibus (GEO, https://www.ncbi.nlm.nih.gov/geo/) under accession GSE144119.

## 5. Conclusions

Our study has provided new insights into the epigenomic and transcriptomic landscapes of CML patients at diagnosis and remission. We have shown that, despite a changing cell composition at major or complete molecular remission, alternative splicing remains aberrantly regulated, with high IR levels affecting multiple cell cycle regulators. Further research is required to test whether sustained RNA processing alterations in CML remission facilitate relapse following TKI cessation. The new knowledge about epigenetic links to alternative splicing in CML could facilitate the development of epigenetic therapeutic adjuvants to increase the likelihood of successful TKI cessation. Modulation of spliceosome function has recently been proposed as a new therapeutic avenue in leukaemia [61], which is an idea reinforced by our results.

## Figures and Tables

**Table 1:** Patient and healthy donor samples used in RNA-seq and WGBS experiments. Sequencing was performed on peripheral blood mononuclear cells (PBMCs).

	Diagnosis	Remission	Control
Number of samples	16	11	6
Sex (F/M)	11/5	8/3	2/4
Median age years (range)	54 (21–69)	56 (23–70)	37 (30–42)
Trial (RESIST/ENESTxtnd/PINNACLE/PERSISTEM)	1/4/4/7	1/4/4/2	
Timepoint	Screening (×12)/Day 1	6–35 months	
First-line TKI (imatinib/nilotinib)	8/8		
RNA-seq and WGBS (RNA-seq only)	10 (+6)	10 (+1)	6

## Supplementary Materials

The following are available online at https://www.mdpi.com/2072-6694/12/12/3738/s1, Data S1: Sample metadata, Date S2: Fusion transcripts, Figure S1: PCA analysis of putative confounders and batch effects, Figure S2: Recurring fusion transcripts, Figure S3: Transcriptomic changes in CML diagnosis and remission, Figure S4: Kinome changes in CML patients from diagnosis to remission, Figure S5: Enriched GO terms and KEGG pathways, Figure S6: Circos plots of DMRs in all of the ten matched patient samples, Figure S7: Multidimensional scaling (MDS) plots of DNA methylation data, Figure S8: Alternative splicing analysis, Figure S9: IR frequencies in subsampled sequencing data, Figure S10: Differential IR of paired diagnosis and remission samples of individual patients, Figure S11: Clustering of intron retention profiles, Figure S12: GO analysis of differential intron-retaining genes, Figure S13: Characteristics of retained introns in CML, Figure S14: Intronic GC content, Figure S15: Expression of transcription elongation factors, Figure S16: CpG methylation around intron splice sites, Figure S17: Expression of RNA binding proteins with enriched binding motifs near frequently retained introns, Figure S18: ChIP-seq data of histone modifications in K562 cells, Figure S19: Differential IR in K562 cells treated with Imatinib or DMSO, File S1: Supplementary Methods, Table S1: Primer sequences for IR validation, Table S2: Primer sequences for the *CLEC12A/MIR223* fusion validation.
