# Supplementary material for: Widespread Aberrant Alternative Splicing despite Molecular Remission in Chronic Myeloid Leukaemia Patients
Source: Cancers (Basel). 2020 Dec 11;12(12):3738. doi: 10.3390/cancers12123738 (PMC7764299; doi:10.3390/cancers12123738)
Supplement: Supplementary file 1 [file cancers-12-03738-s001.zip › cancers-997156-suppl-final/Supplementary files/File S1.docx]

Supplementary Materials

Widespread Aberrant Alternative Splicing despite Molecular Remission in Chronic Myeloid Leukaemia Patients

Ulf Schmitz, Jaynish S. Shah, Bijay P. Dhungel, Geoffray Monteuuis, Phuc-Loi Luu, Veronika Petrova, Cynthia Metierre, Shalima S. Nair, Charles G. Bailey, Verity A. Saunders, Ali G. Turhan, Deborah L. White, Susan Branford, Susan J. Clark, Timothy P. Hughes, Justin J.-L. Wong and John E.J. Rasko

Supplementary Methods

Intron retention quantification

The IR-ratio is a measure of intron inclusion in mature mRNA transcripts, i.e. the ratio of the median read coverage of the intron to that of its flanking exons. We refer to this measure as the “IR ratio,” while others have also used the term “percent spliced in” (PSI) [1]. The IR ratio is in the range of 0–1; however, we only considered introns with an IR ratio ≥ 0.1 as biologically meaningful. We excluded introns with insufficient splicing depth (<4 reads correctly crossing the splice junction) and insufficient coverage (splicing depth + trimmed mean intron depth < 10). Different filters were applied for pooled samples in unique conditions (intron coverage ≥ 75%; intron depth ≥ 10; splices left ≥ 50; IR ratio ≥ 0.1).

IRFinder has a built-in routine to handle confounding factors. For example, partial IR resulting from splicing inside the intron is a distinct process and was not considered. On a per-gene basis, we considered the highest observed IR ratio for any of the retained introns as the gene’s IR ratio.

Differential methylation and IR

For differential methylation analysis in and around retained/non-retained introns, the raw counts of methylated cytosines and of the total coverage were aggregated into sliding windows (width: 10 bp, step-size: 1 bp) spanning across the 5′ and 3′ splice sites (+/− 100 bp) and the intron body. Only CpG sites with the coverage of 5 or more reads were considered.

DNA methylation was modelled as count data using the Generalised Linear Mixed Model (GLMM), assuming a binomial distribution for the response variable

$$Methylation Ratio \sim B\left( n_{i},p_{i} \right)$$

Where $p_{i}$ is a probability of a CpG site to be methylated and $n_{i}$ is the total number of reads mapped to a CpG site. The data was fit into the following model

$$logit\left( \frac{p_{ij}}{1- p_{ij}} \right)=\beta_{0}+\beta_{T}T_{j}+{T_{j}W}_{j}+I_{j}+I_{j}P_{j}+\varepsilon_{ij}$$

Where a vector $T_{j}$, indicating the intron type (1: retained, 0: non-retained), represents fixed effect. Random effects included:

$W_{j}$—vector indicating a distance from a reference point (either 5′, 3′ splice site or intron body),

$I_{j}$—vector containing individual intron identifiers,

$P_{j}$—vector indicating patient samples.

The GLMM was fitted using the glmer function in the lme4 package (github.com/lme4/lme4/). Multiple comparison testing was implemented with the Scheffe method in the multcomp package (multcomp.r-forge.r-project.org) using the glht function.

ChIP-seq data analysis

Processed ChIP-seq data mapped to GRCh38 for K562 cells were downloaded from the ENCODE portal (encodeproject.org) in ‘narrowPeak’ format. Only ‘replicated peaks’ were used in subsequent analyses. Accession numbers and number of biological replicates were as follows: H3K9ac (ENCFF667VWL, ENCFF558JOB and ENCFF306MNO; *n* = 3), H3K36me3 (ENCFF053DAC and ENCFF631VWP; *n* = 2) and H3K4me1 (ENCFF159VKJ; *n* = 1). Ten-time bootstrapping was performed with the R package boot (bootsize of 100). For each boot, both retained and non-retained introns were first split into expression quartiles based on the number of reads that mapped across the 3′ and 5′ flanking exons, which we used as a surrogate for gene expression. The number of ChIP-seq peaks mapping to retained and non-retained introns were reported and data was plotted with mean ± standard error.

Fusion gene validation

The *CLEC12A-MIR223* fusion was validated with PCR using primers listed in Table S2. Forward primer binding to *CLEC12A* and reverse primer binding to *MIR223* were designed based on the reads from the RNA sequencing data. qRT-PCR was then performed on matched diagnosis and remission samples as described in *IR validation*. For quantification of mature *miR-223* levels, MystiCq MicroRNA Quantitation System (MIRRT, Sigma Aldrich) was used as per the manufacturer’s protocol. In short, cDNA was synthesized from 1 𝜇g of Turbo DNase treated RNA and qRT-PCR was performed using primers for *miR-223* (MIRAP00282, Sigma Aldrich) and MystiCq Universal PCR primer (MIRUP). Expression levels of *miR-223* were then normalized to the expression level of *RNU6-1* (MIRCP00001).

Reference

1. Pimentel H, Parra M, Gee SL, Mohandas N, Pachter L, Conboy JG. A dynamic intron retention program enriched in RNA processing genes regulates gene expression during terminal erythropoiesis. *Nucleic Acids Res*. 2016;44(2):838-851.

**Publisher’s Note:** MDPI stays neutral with regard to jurisdictional claims in published maps and institutional affiliations.

| 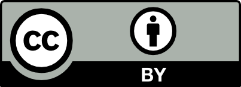 | © 2020 by the authors. Licensee MDPI, Basel, Switzerland. This article is an open access article distributed under the terms and conditions of the Creative Commons Attribution (CC BY) license (http://creativecommons.org/licenses/by/4.0/). |
| --- | --- |
