# Supplementary material for: Widespread Aberrant Alternative Splicing despite Molecular Remission in Chronic Myeloid Leukaemia Patients
Source: Cancers (Basel). 2020 Dec 11;12(12):3738. doi: 10.3390/cancers12123738 (PMC7764299; doi:10.3390/cancers12123738)
Supplement: Supplementary file 1 [file cancers-12-03738-s001.zip › cancers-997156-suppl-final/Supplementary files/Supplementary Materials.docx]

Supplementary Materials

Widespread Aberrant Alternative Splicing despite Molecular Remission in Chronic Myeloid Leukaemia Patients

Ulf Schmitz, Jaynish S. Shah, Bijay P. Dhungel, Geoffray Monteuuis, Phuc-Loi Luu, Veronika Petrova, Cynthia Metierre, Shalima S. Nair, Charles G. Bailey, Verity A. Saunders, Ali G. Turhan, Deborah L. White, Susan Branford, Susan J. Clark, Timothy P. Hughes, Justin J.-L. Wong and John E.J. Rasko

Supplementary Figure 1
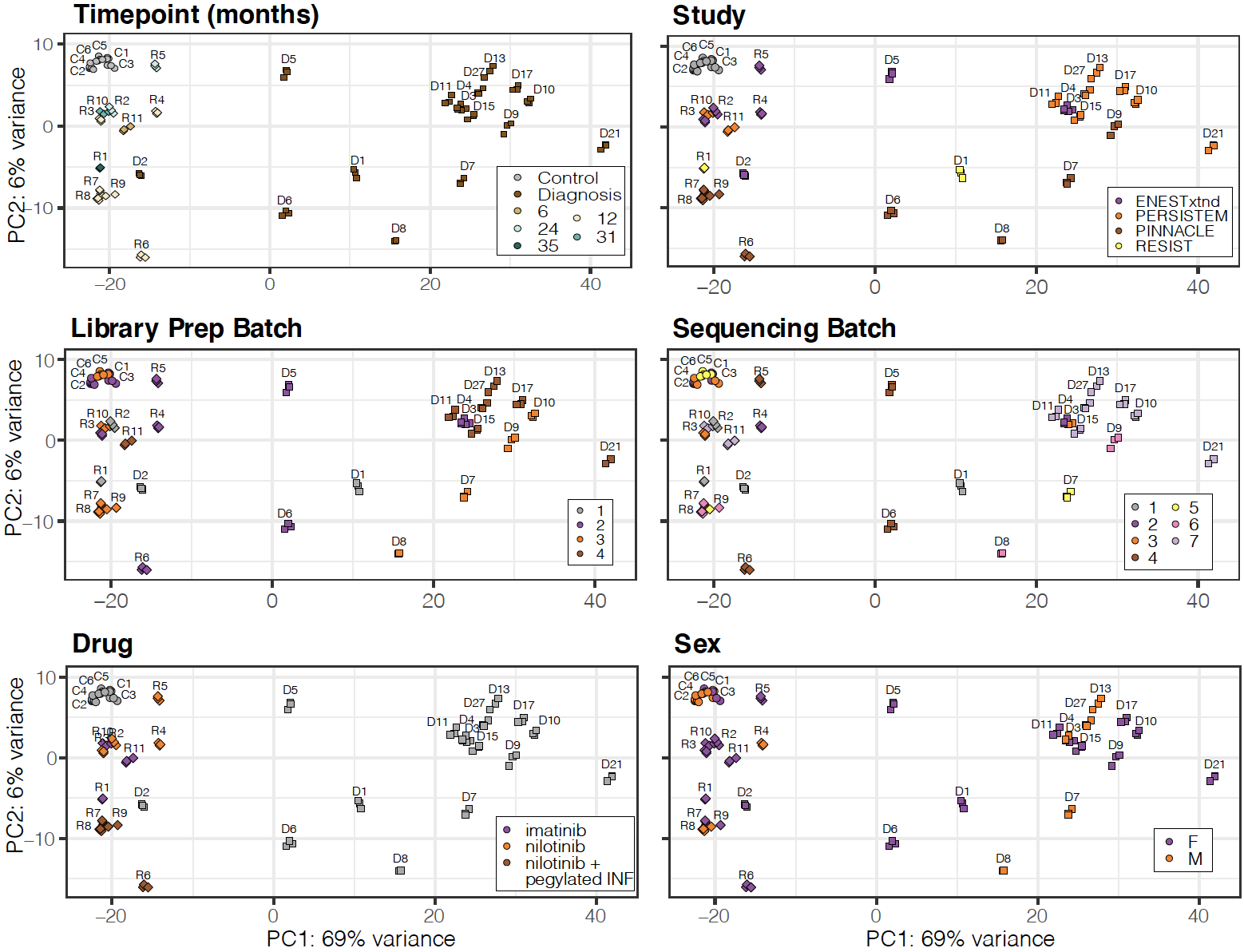


**Figure S1.** PCA analysis of putative confounders and batch effects. Each subfigure illustrates the distribution of putative confounders and batch effects in a principal component analysis. The results suggest that trial, library preparation and sequencing batch, treatment regimen, or sex have no impact on the transcriptomic profiles observed.


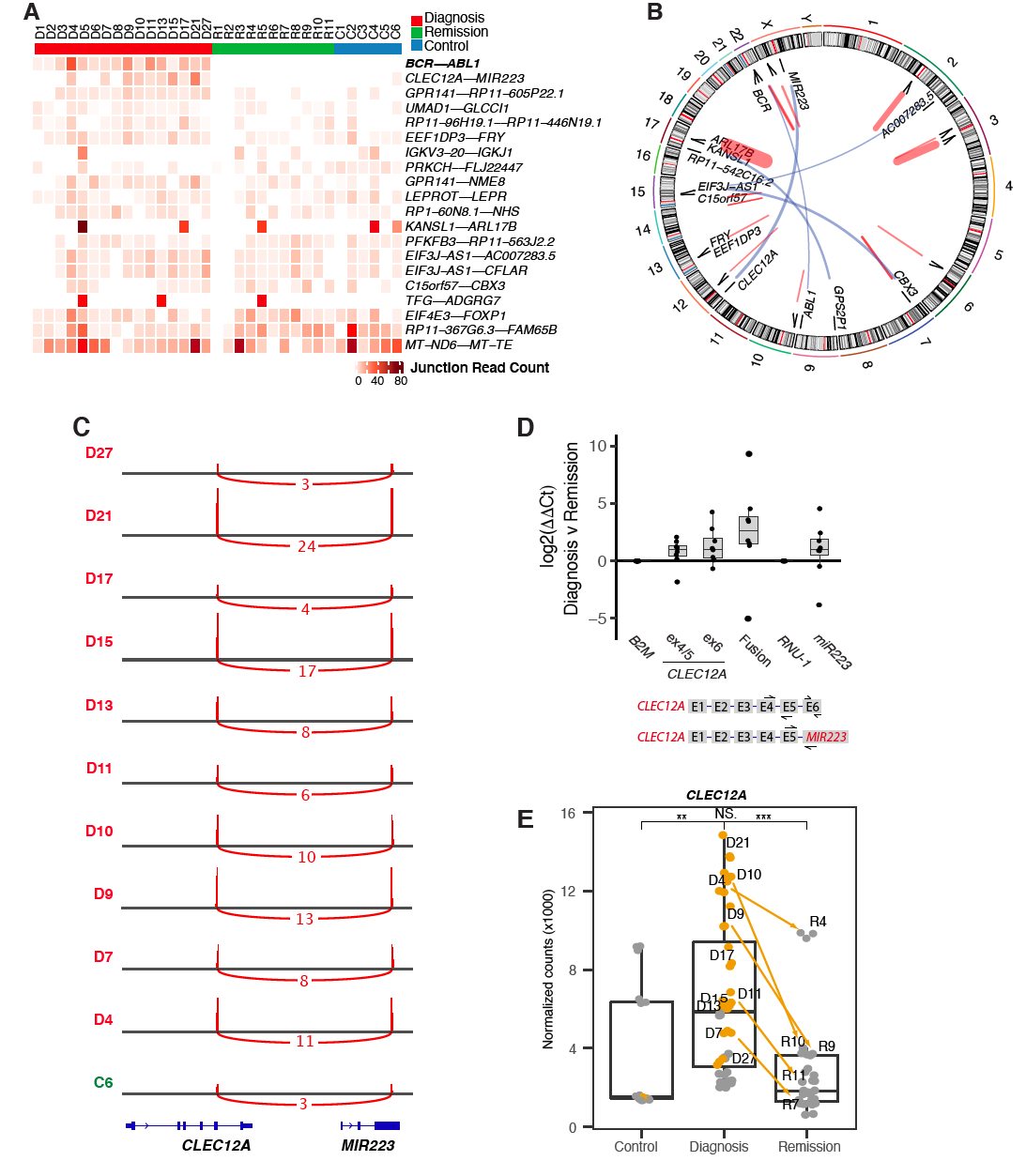


**Figure S2.** Recurring fusion transcripts. (**A**) Recurring fusions transcripts in diagnosis (**D**), remission (R), and control (**C**) samples. Shown are only those with a total junction read count >50. Fusions discussed in the manuscript are highlighted in bold. (**B**) The Circos plot illustrates inter- and intra-chromosomal fusions (blue and red lines, respectively). The line width correlates with the read depth supporting each fusion. Only inter-chromosomal fusions are labeled. A summary of all identified fusions is provided in Data S2. Fusion genes have been identified using STAR-FUSION (STAR-Fusion.github.io); the circos plot was generated using the chimeraviz Bioconductor package [1]. (**C**) Read counts that span across the fusion breakpoints in 10 diagnosis (red) and 1 control sample (green). The gene structure is collated from all isoforms of *CLEC12A* and *MIR223*. In all *CLEC12A*-*MIR223*-positive samples the fusion breakpoints are at the end of exon 5 in *CLEC12A* (chr12:9982129+) and at the 3′ end of the last exon (exon 3) of *MIR223* (chrX:66020127+). (**D**) Expression fold changes (∆∆Ct) of *CLEC12A*, the *CLEC12A*-*MIR223* fusion transcript, and *miR-223*. Expression of the major *CLEC12A* isoform was determined based on primer pairs in exon 4/5 and 6. Exon 6 is not part of the fusion transcript. Primer sequences are provided in Table S1. (**E**) Normalized counts of *CLEC12A* across samples. Orange arrows indicate matched samples.


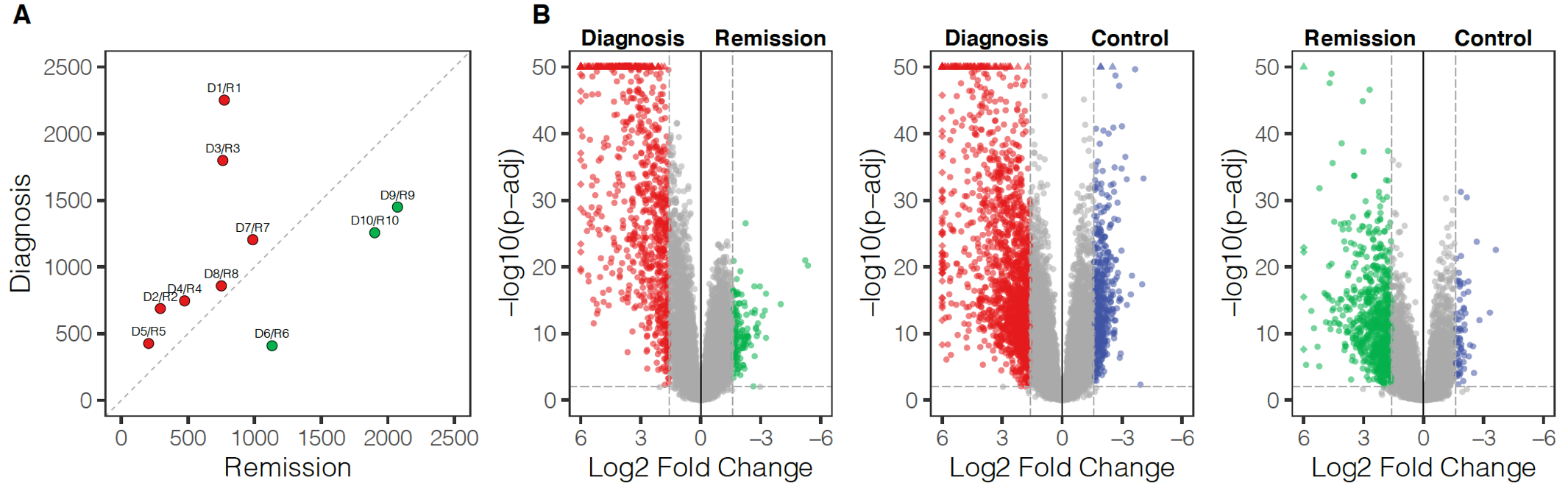


**Figure S3.** Transcriptomic changes in CML diagnosis and remission. (**A**) Number of differentially expressed genes (|*FC| ≥* 3) in each patient at diagnosis vs remission. (**B**) Volcano plots illustrating differentially expressed genes in paired comparisons of all diagnosis/remission/control samples (upregulated in: diagnosis—red, remission—green, control—blue; |*FC| ≥* 3*; p-adj. <* 0.05).


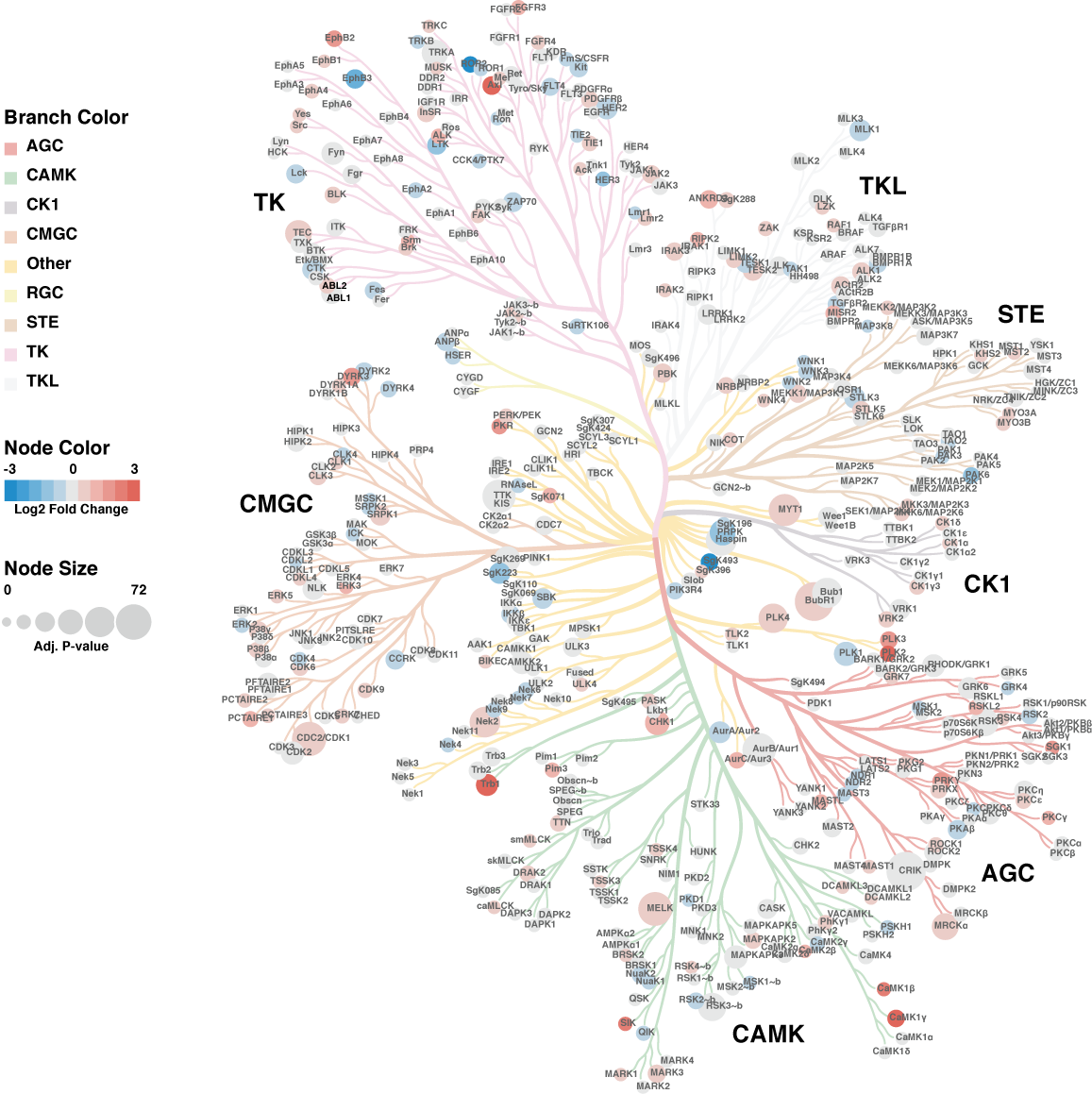


**Figure S4.** Kinome changes in CML patients from diagnosis to remission. Differential gene expression in the human kinome. The kinome tree was generated using the interactive web application Coral (http://phanstiel-lab.med.unc.edu/Coral). AGC—protein kinase A, G, and C families; CAMK—Calmodulin/Calcium-regulated kinases; CK1—Cell Kinase 1 family; CMGC—CDK, MAPK, GSK3 and CLK families; RGC—Receptor Guanylate Cyclases; STE—homologs of the yeast STE7, STE11 and STE20 genes; TK—tyrosine kinases; TKL—tyrosine kinase-like.


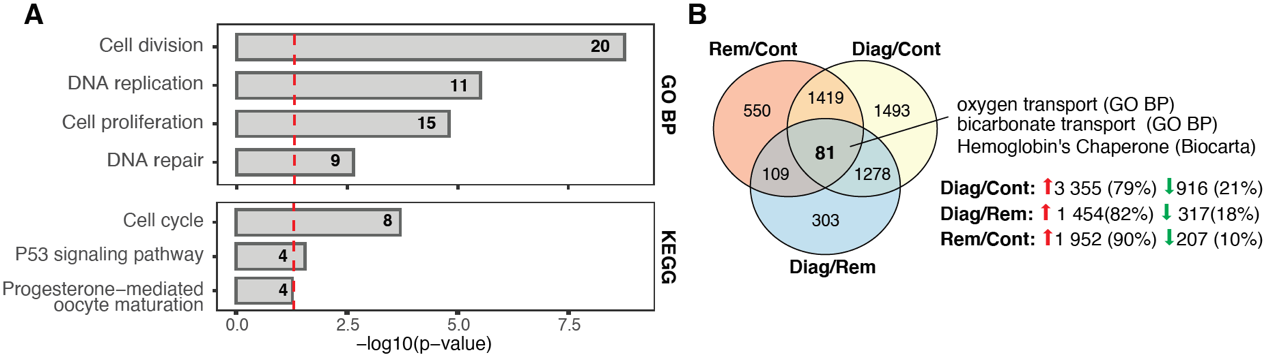


**Figure S5.** Enriched GO terms and KEGG pathways. (**A**) Enriched GO terms and KEGG pathways associated with differentially expressed genes (remission vs diagnosis). Numbers in bars indicate how many differentially expressed genes are associated with each term or pathway; the red dashed line marks *p* = 0.05. (**B**) Venn diagram illustrating intersections of differentially expressed genes. Genes with absolute fold change |*FC| ≥* 3 and *p-adj. ≤* 0.05 (Wald test) were considered differentially expressed. GOBP: gene ontology biological process.


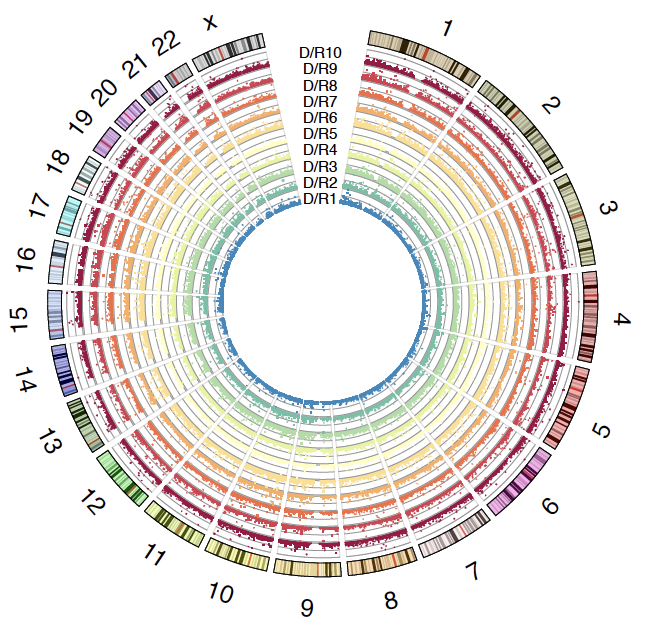


**Figure S6.** Circos plots of DMRs in all of the ten matched patient samples.

Supplementary Figure 7


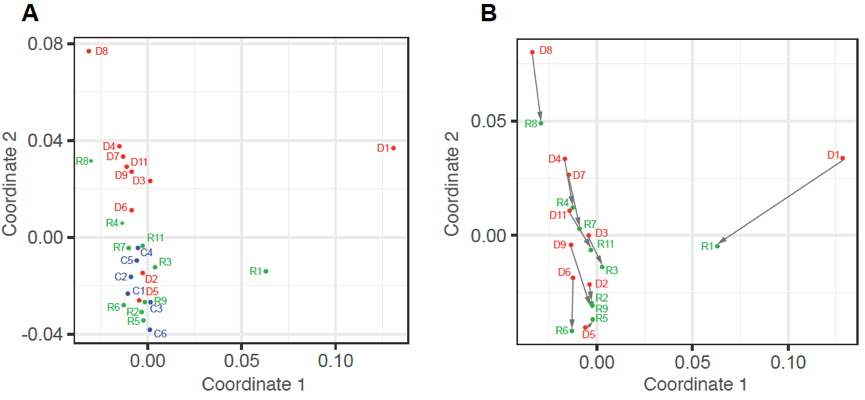


**Figure S7.** Multidimensional scaling (MDS) plots of DNA methylation data. (**A**) Diagnosis (red), Remission (blue), Normal (green). (**B**) Methylation changes in matched samples. Arrows indicate pairs of matched diagnosis and remission samples.


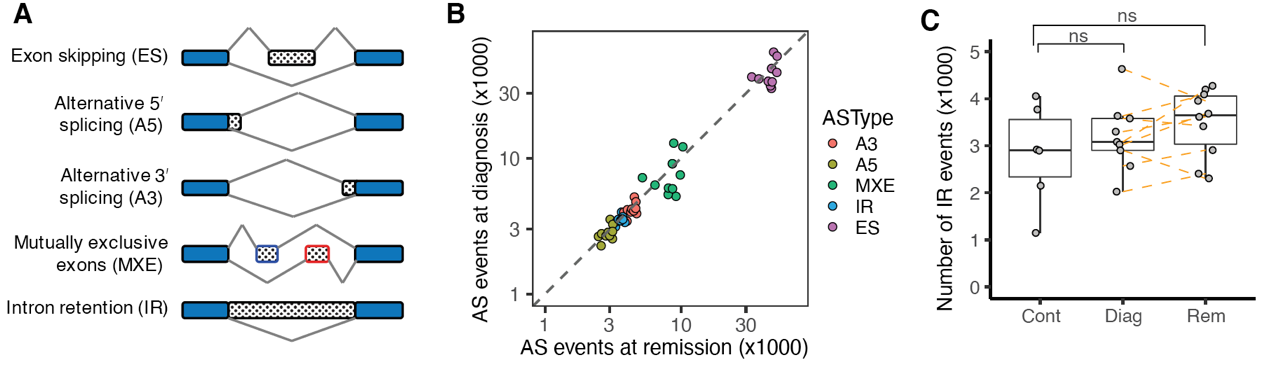


**Figure S8.** Alternative splicing analysis. (**A**) Schematic of different forms of alternative splicing analysed. (**B**) The frequency of alternative splicing events in matched diagnosis/remission samples of CML patients. Event frequency was determined using rMATS [2]. (**C**) IR frequencies in normal control, diagnosis, and remission samples. Yellow dashed lines connect matched diagnosis/remission samples.


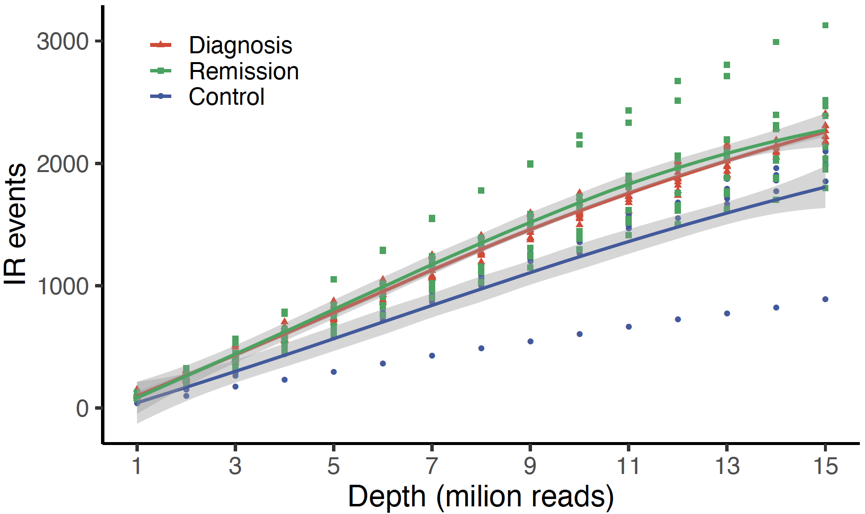


**Figure S9.** IR frequencies in subsampled sequencing data. Kolmogorov-Smirnov tests showed that the number of IR events in diagnosis and remission subsamples are larger than in control subsamples with *p* = 0.035 and *p* = 0.013, respectively.


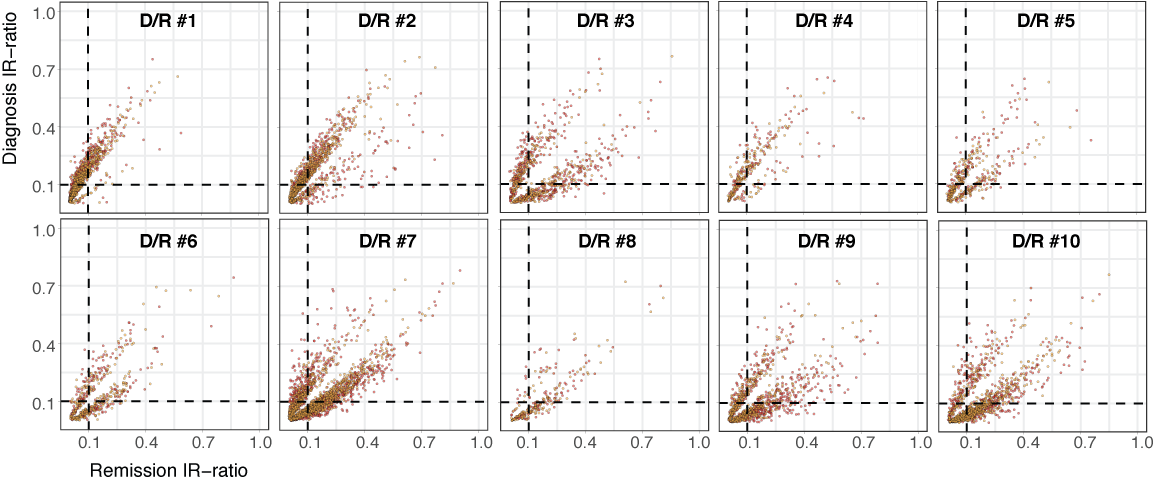


**Figure S10.** Differential IR of paired diagnosis and remission samples of individual patients. The scatter plots illustrate the IR ratios of significantly differentially retained introns in 10 paired diagnosis/remission samples (Audic and Claverie test; yellow: *p-adj. ≤* 0.05; red: *p-adj. ≤* 0.01).


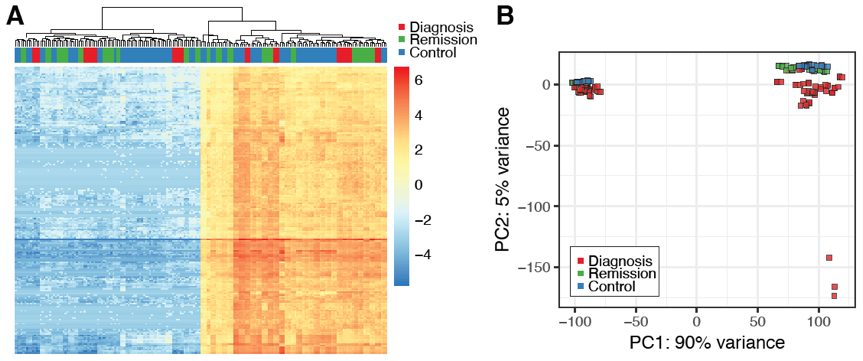


**Figure S11.** Clustering of intron retention profiles. (**A**) Heatmap of top 200 most dynamic IR events. (**B**) PCA plot of IR profiles.


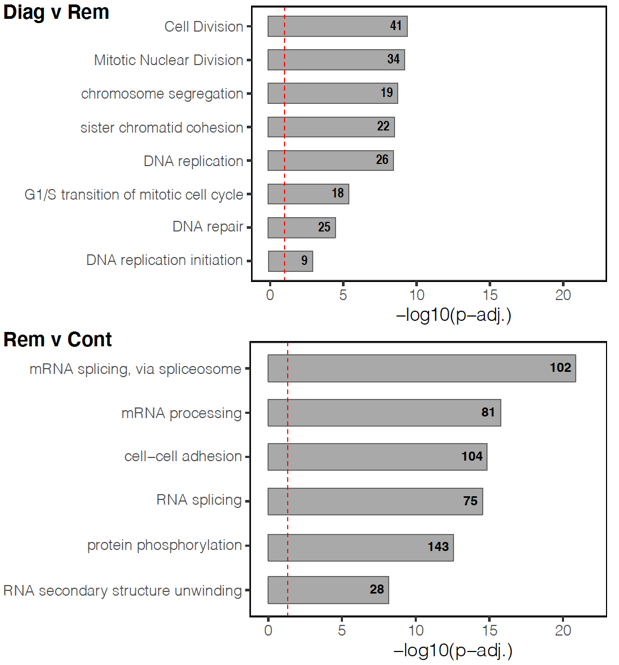


**Figure S12.** GO analysis of differential intron-retaining genes. Numbers in bars indicate how many differentially expressed genes are associated with each term; the red dashed line marks *p* = 0.05.


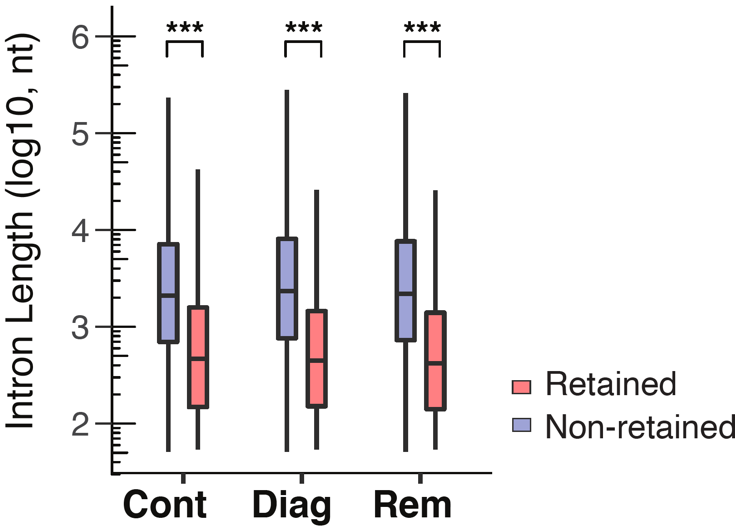


**Figure S13.** Characteristics of retained introns in CML. Lengths of non-retained (blue) and retained (red) introns in normal controls, at diagnosis, and remission. Mann-Whitney U test; *** *p*-value < 2.2 × 10^−16^.


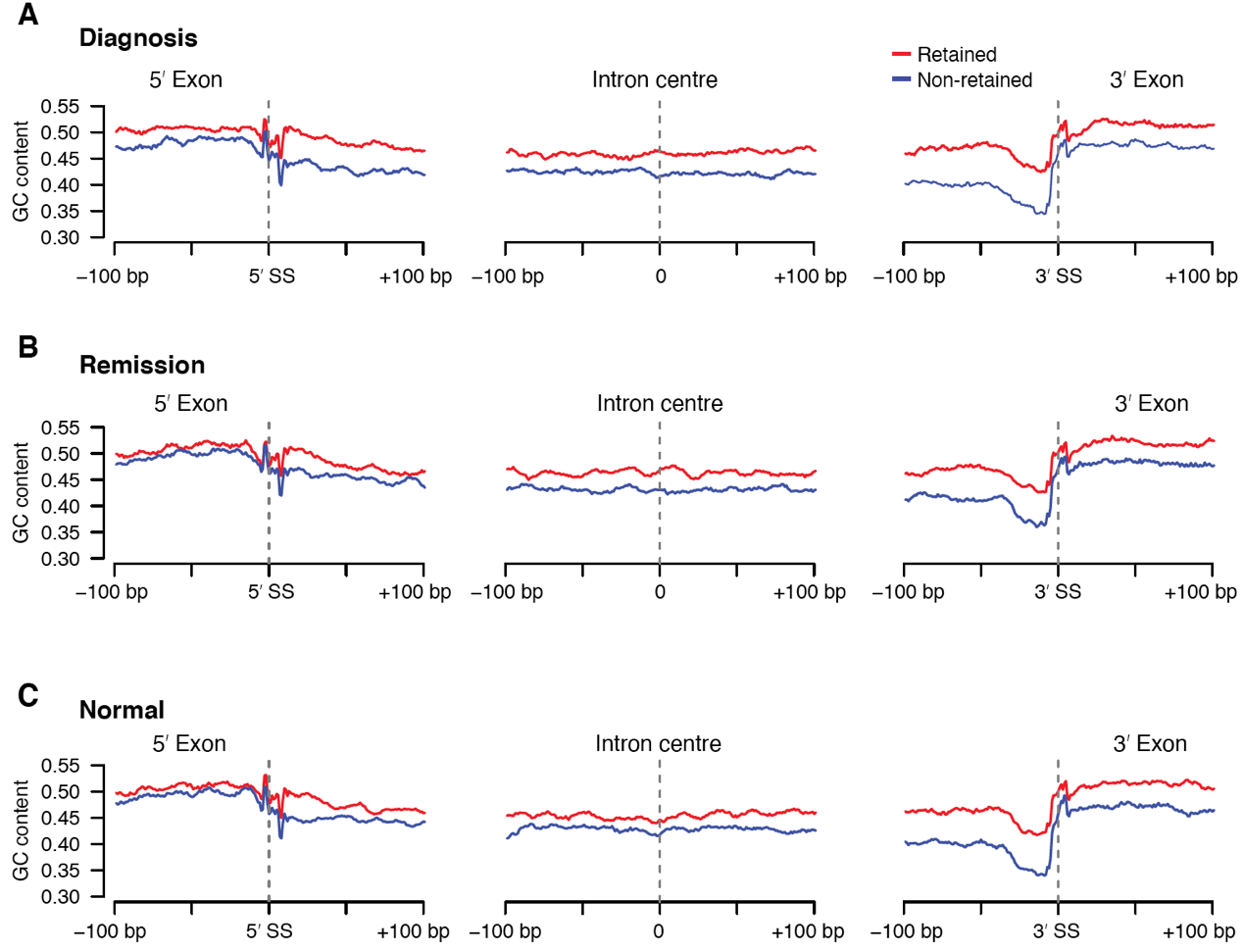


**Figure S14.** Intronic GC content. Intronic GC content of 1,000 randomly selected non-retained (blue) and retained (red) introns in diagnosis (**A**), remission (**B**), and control samples (**C**). The lines extend 100 bp into the flanking exons, 100 bp on both ends of the intron and 200 bp in the intron centre. Mann-Whitney U test; *p*-value < 2.2 × 10^−16^.


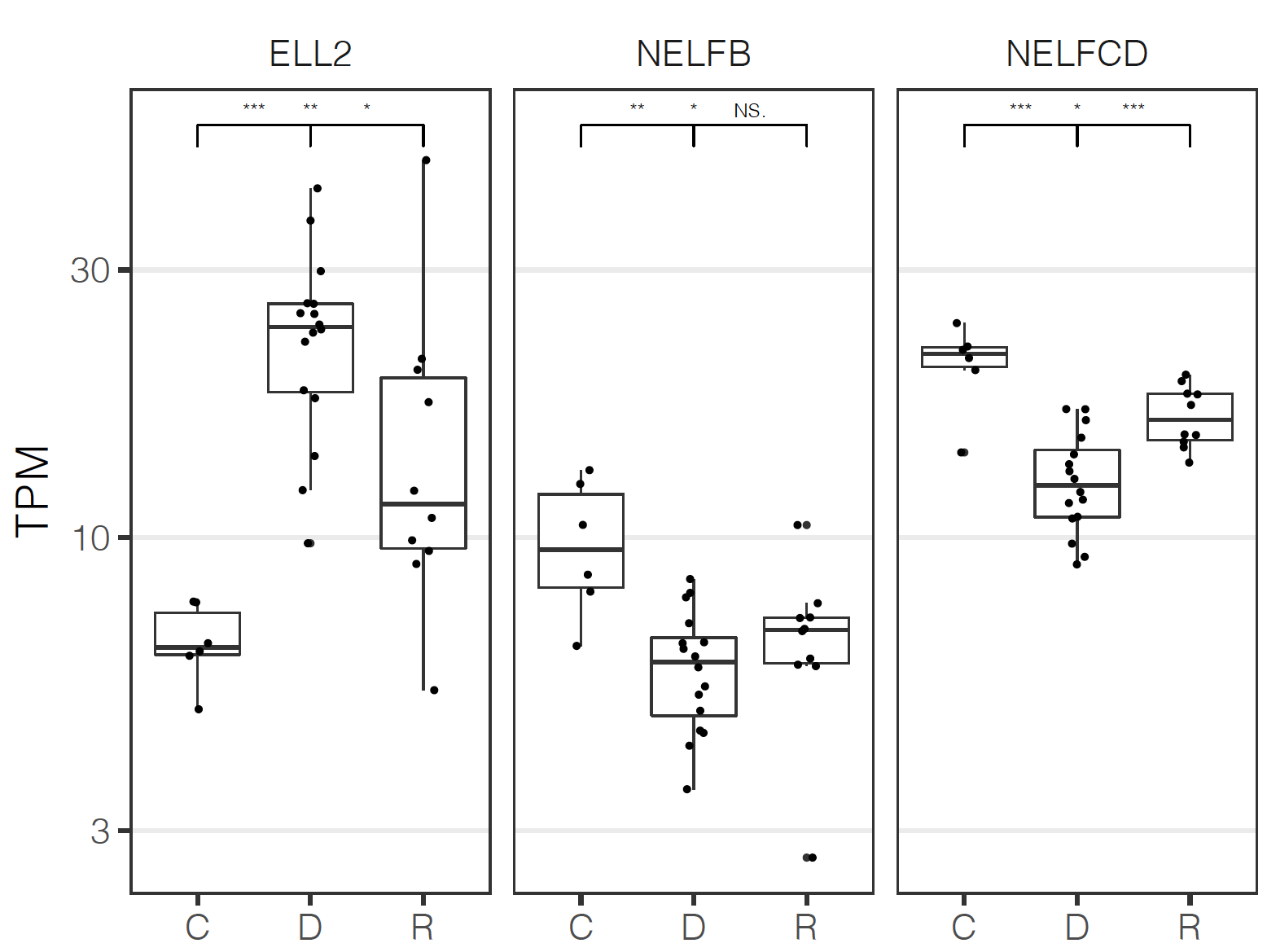


**Figure S15.** Expression of transcription elongation factors. TPM—transcripts per million mapped reads; C—control; D—diagnosis; R—remission; NS.—non-significant, * *p* < 0.05, ** *p* < 0.01, *** *p* < 0.001.


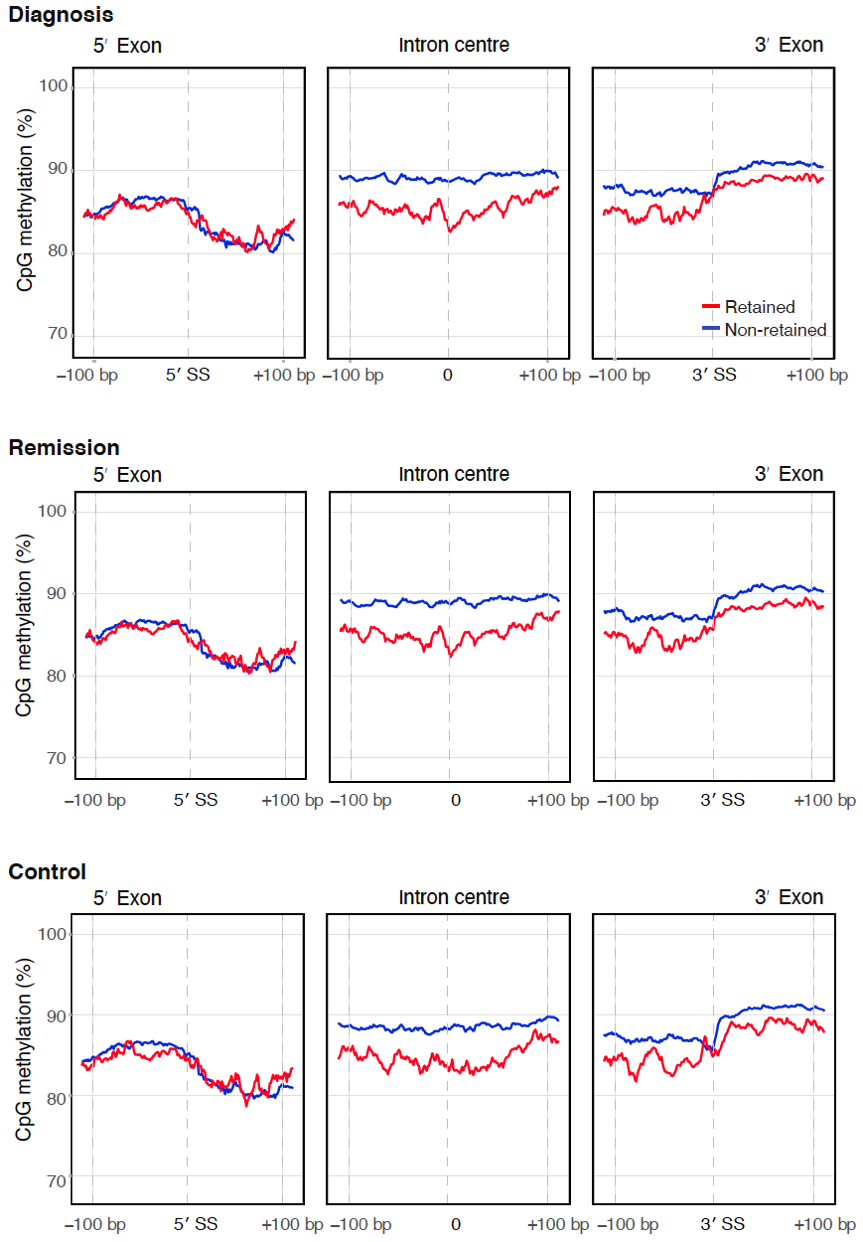


**Figure S16.** CpG methylation around intron splice sites (± 200 bp) and in the centre (200 bp) of retained- (red) and non-retained introns (blue) in CML diagnosis, remission and control samples.


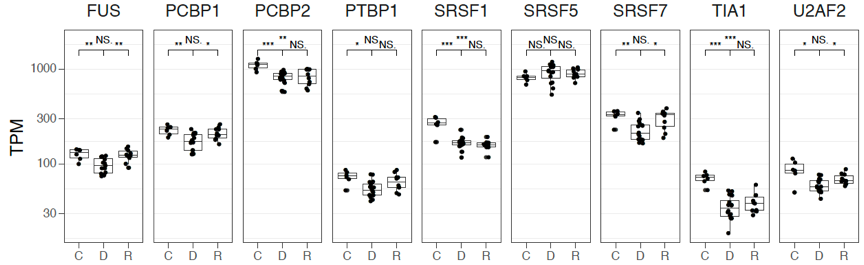


**Figure S17.** Expression of RNA binding proteins with enriched binding motifs near frequently retained introns [3]. TPM—transcripts per million mapped reads; C—control; D—diagnosis; R—remission; NS.—non-significant, * *p* < 0.05, ** *p* < 0.01, *** *p* < 0.001.


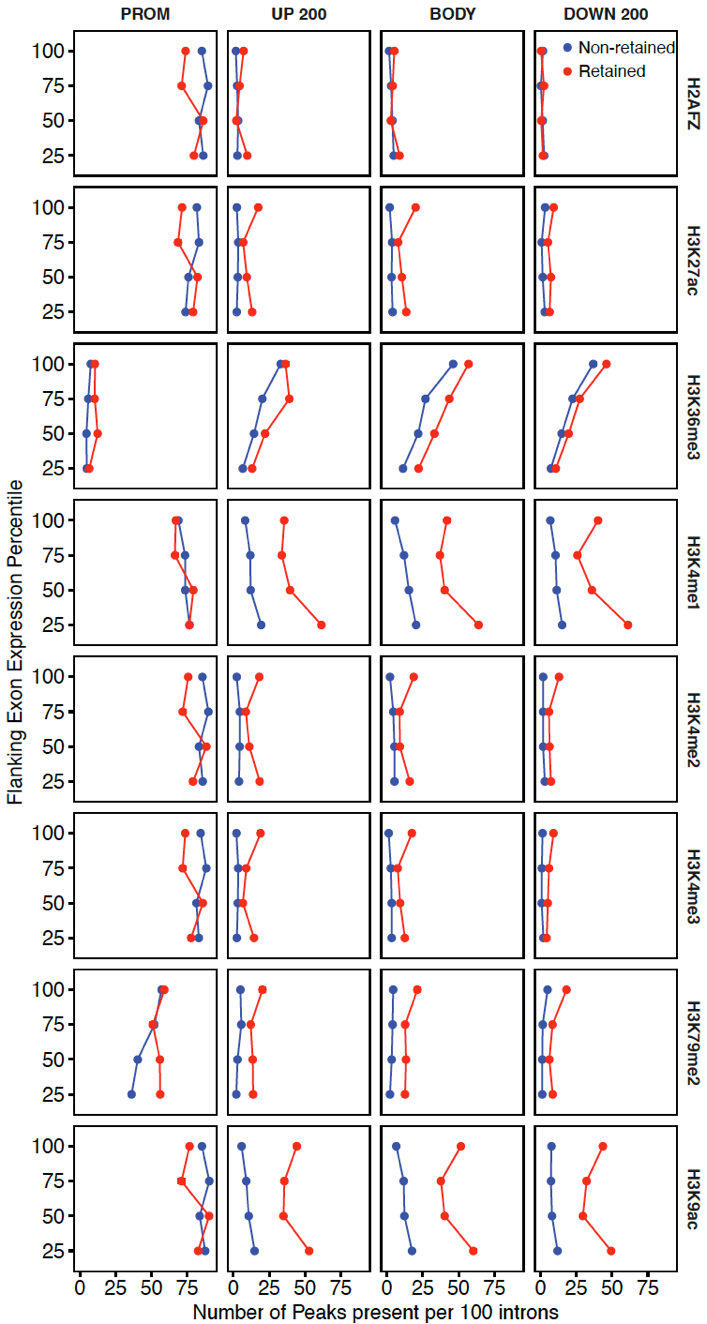


**Figure S18.** ChIP-seq data of histone modifications in K562 cells. Numbers of peaks for histone marks in the promoter region (PROM), 200 nt up- and downstream and within retained (red) and non- retained (blue) introns. The analysis was repeated for each expression quartile to exclude the possibility that increased histone modifications are associated with gene expression.


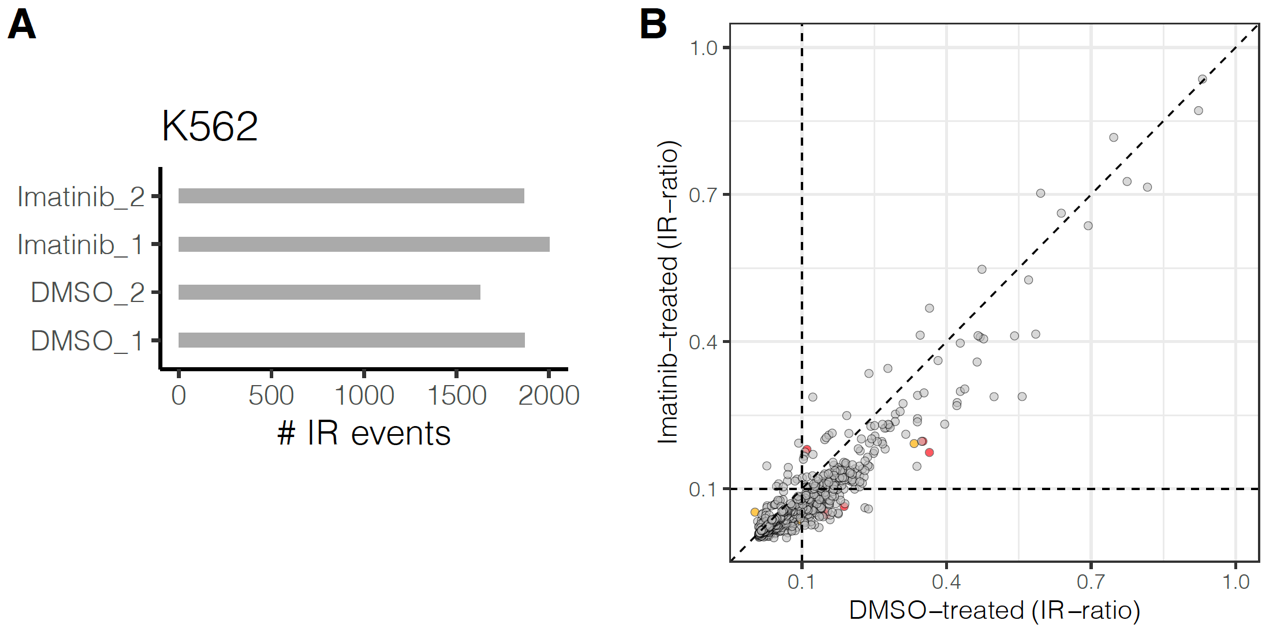


**Figure S19.** Differential IR in K562 cells treated with Imatinib or DMSO. (**A**) Number of IR events in K562 cells treated with imatinib or DMSO. (**B**) The scatter plot illustrates the IR ratios of differentially retained introns in K562 cells treated with imatinib or DMSO (Audic and Claverie test; grey: *p-adj*. > 0.05; yellow: *p-adj*. ≤ 0.05; red: *p-adj*. ≤ 0.01). RNA-seq data was retrieved from GEO accession: GSM2823720.

**Table S1.** Primer sequences for IR validation.

| **Gene** | **Forward/Reverse** | **Feature** | **Primer Sequence** |
| --- | --- | --- | --- |
| *CKS2* | F | Exon 1 | CAGTTGTAGAAAAGGCACTGGA |
| *CKS2* | R | Intron 1 | CCAGCCTAGACTCTGTTGGA |
| *CKS2* | F | Exon 1 | CACTACGAGTACCGGCATGTT |
| *CKS2* | R | Exon 2 | GGACACCAAGTCTCCTCCAC |
| *CCL3* | F | Exon 1 | GCAGCAGACAGTGGTCAGTC |
| *CCL3* | R | Intron 1 | CAGACTCACGTGATGCAGAGA |
| *CCL3* | F | Exon 1 | GCATCACTTGCTGCTGACAC |
| *CCL3* | R | Exon 2 | TGGCTGCTCGTCTCAAAGTA |
| *SERPINB1* | F | Exon 2 | TCCAAGGTCAGCAGAAACAA |
| *SERPINB1* | R | Intron 2 | TGGAAGCCTGCATACAAGTG |
| *SERPINB1* | F | Exon 2 | CAGCTGTCCAAGACTTTCCA |
| *SERPINB1* | R | Exon 3 | GAATATAAGACGCTCCACGTTTG |

**Table S2.** Primer sequences for the *CLEC12A/MIR223* fusion validation.

| **Gene** | **Forward/Reverse** | **Feature** | **Primer Sequence** |
| --- | --- | --- | --- |
| *CLEC12A* | F | Exon6 | TGCCAGAGGCAACATCAAA |
| *CLEC12A* | R | Exon6 | CTATACTTCCATGGGACTCCCT |
| *CLEC12A/MIR223* | F | Fusion | GGCTGGGATTATCTCCTGAAG |
| *CLEC12A/MIR223* | R | Fusion | TTAGGCAGATGGCTGGTTG |
| *CLEC12A* | F | Exon 4 | ATGCCAGCCTGTTGAAGATAA |
| *CLEC12A* | R | Exon 5 | CAGGAGATAATCCCAGCCAATAG |

References

1. Lagstad, S.; Zhao, S.; Hoff, A.M.; Johannessen, B.; Lingjaerde, O.C.; Skotheim, R.I. chimeraviz: a tool for visualizing chimeric RNA. *Bioinformatics*. **2017**, *33*: 2954–2956. doi: 10.1093/bioinformatics/btx329
2. Shen, S.; Park, J.W.; Lu, Z.X.; Lin, L.; Henry, M.D.; Wu, Y.N.; Zhou, Q.; Xing, Y. rMATS: robust and flexible detection of differential alternative splicing from replicate RNA-Seq data. *Proc. Natl. Acad. Sci. USA*. **2014**, *111*, E5593–5601. doi: 10.1073/pnas.1419161111
3. Middleton, R.; Gao, D.; Thomas, A.; Singh, B.; Au, A.; Wong, J.J.-L.; Bomane, A.; Cosson, B.; Eyras, E.; Rasko, J.E.J.; et al. IRFinder: assessing the impact of intron retention on mammalian gene expression. *Genome Biol*. **2017**, *18,* 51. doi: 10.1186/s13059-017-1184-4.

**Publisher’s Note:** MDPI stays neutral with regard to jurisdictional claims in published maps and institutional affiliations.

| 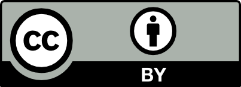 | © 2020 by the authors. Licensee MDPI, Basel, Switzerland. This article is an open access article distributed under the terms and conditions of the Creative Commons Attribution (CC BY) license (http://creativecommons.org/licenses/by/4.0/). |
| --- | --- |
